# Toeplitz-Enhanced Array Covariance Processing for DOA Estimation Under Low SNR and Limited Snapshots

**DOI:** 10.3390/s26185803

**Published:** 2026-09-13

**Authors:** Xin Jin, Yanan Fan, Xiujuan Yao, Xinyu Li, Yanan Meng, Yi Yan, Xiang Gao

**Affiliations:** 1 National Space Science Center, Chinese Academy of Sciences, Beijing 100190, China; jinxin231@mails.ucas.ac.cn (X.J.); yaoxj@nssc.ac.cn (X.Y.); lixinyu24@mails.ucas.ac.cn (X.L.); mengyanan24@mails.ucas.ac.cn (Y.M.); yanyi@nssc.ac.cn (Y.Y.); gaoxiang@nssc.ac.cn (X.G.); 2 School of Electronic, Electrical and Communication Engineering, University of Chinese Academy of Sciences, Beijing 100049, China

**Keywords:** direction-of-arrival estimation, low signal-to-noise ratio, limited snapshots, Toeplitz structure constraint, array signal processing

## Abstract

Multiple-source DOA (Direction of Arrival) estimation is vital for array processing, radar, and integrated sensing and communications, yet classical subspace methods degrade under low signal-to-noise ratios and snapshot-starved conditions due to inaccurate sample covariance matrices. To address this, we propose a Toeplitz-enhanced neural network (TENN-DOA) for DOA estimation, a hybrid physics-informed framework for uniform linear arrays that combines the array signal processing prior with a lightweight learning-based regressor. The front end explicitly enforces the Hermitian–Toeplitz structure and fuses the projected matrix with the sample covariance via an analytically derived optimal shrinkage coefficient, yielding a robust covariance estimate. This enhanced representation is mapped onto an overcomplete angular dictionary, producing a feature sequence structurally coupled with the array manifold. A pooling-free one-dimensional convolutional neural network with decreasing kernel sizes starts with large kernels to capture the broad spectral envelope from grid mismatch, and then regresses to a pseudo spatial spectrum under multi-hot supervision for grid-point estimates. The Monte Carlo simulation results show that under the conditions of low signal-to-noise ratio, limited snapshots and the simulated ideal uniform linear array scenario, TENN-DOA achieves a higher resolution probability and lower root mean square error compared with MUSIC, TLS-ESPRIT and the deep learning-based baseline algorithm DA-MUSIC.

## 1. Introduction

Direction-of-arrival (DOA) estimation infers the directions of incident waves from array measurements and is a basic operation in radar, communications, sonar, electronic reconnaissance, and space situational awareness. Its accuracy is governed by the array calibration, the signal-to-noise ratio (SNR), the number of snapshots, and the quality of covariance estimation [1,2]. Classical methods, such as MUSIC and ESPRIT, use the eigen structure of the sample covariance matrix (SCM) to separate signal and noise subspaces and can provide high resolution when the array is well calibrated, the SNR is adequate, and enough snapshots are available [3,4,5]. Passive sensing, however, often observes intermittent or non-cooperative sources over short coherent processing intervals. The resulting low SNR and limited snapshot regimes make covariance estimation the first bottleneck rather than a minor implementation detail.

With few snapshots, the SCM can be poorly conditioned, rank deficient, or strongly perturbed relative to the population covariance. The associated eigenvalue gap and subspace orthogonality become less reliable, so spectral peaks broaden, merge, or develop spurious maxima. This threshold behavior is well documented for covariance-based estimators [6,7]. Linear shrinkage provides a principled way to reduce the covariance–variance by combining the SCM with a structured target; its benefit is strong when the target reflects reliable prior information, but its coefficient remains model and distribution dependent [8,9].

For an ideal calibrated ULA with spatially stationary statistics, the population co-variance is Hermitian–Toeplitz: entries on the same diagonal have the same expectation. Toeplitz reconstruction therefore provides a physically motivated regularization. Dai et al. showed that Toeplitz approximation can improve covariance-based DOA estimation for coherent sources in impulsive noise environments [10]. In the present work, the Toeplitz projection serves as the target of a data-adaptive shrinkage step; it is a structured pre-processing choice rather than a claim that the Toeplitz projection alone resolves all nonideal array effects.

Sparse and continuous-domain estimators provide complementary alternatives. Grid-based sparse recovery formulates DOA estimation as a regularized inverse problem, whereas atomic norm methods operate in a continuous parameter domain. Adaptive Lp minimization, UAMP with a Bernoulli–Gaussian prior, and reweighted atomic norm minimization are representative examples [11,12,13]. Their performance and computational cost depend on the regularization, stopping criteria, dictionary resolution, and off-grid treatment. We therefore include SBL and off-grid SBL as explicit comparison baselines. Sparse array design offers another route to increased degrees of freedom: nested and coprime arrays extend the virtual aperture through difference arrays [14,15], while recent designs address irregular sparse arrays and near-field configurations [16,17]. While array design is vital for resolution improvement, its performance depends on the geometry, array continuity, and covariance estimation accuracy. Algorithmic solutions are still needed for legacy ULA systems with fixed hardware.

Deep learning has also been used for robust, sparse array, gridless, and efficient DOA estimation. Prior work includes neural estimators robust to array imperfections [18], low SNR multilabel prediction [19], coarse-to-fine learning for coprime arrays with sensor position errors [20], knowledge-enhanced learning for frequency-hopping signals [21], sparse prior CNNs [22], complex-valued gridless networks [23], sparse array covariance transformation [24], subspace representation learning [25], residual CNNs for 2D DOA [26], SVM–random forest regression [27], and zero-padded DFT estimators [28]. Collectively, these studies show that deep networks can learn nonlinear array features inaccessible to linear models; however, their performance remains highly dependent on the input feature quality and training data distribution.

The specific gap addressed here is the combination of low SNR, limited snapshots, and closely spaced sources in an ideal ULA. Directly feeding a degraded SCM into a network leaves the model to learn the covariance denoising, array geometry encoding, and peak discrimination simultaneously. Conversely, a purely model-driven eigenspace or sparse solver may incur threshold behavior or higher inference cost in the same regime. The closest methodological families are summarized in Table 1: structured covariance reconstruction supplies an analytic prior, sparse prior CNNs provide a learned grid spectrum, complex-valued gridless networks target the continuous output, and DA-MUSIC learns a covariance enhancement before subspace processing [10,22,23,29].

The objective of this study is consequently narrow and testable: to determine whether a Hermitian–Toeplitz/shrinkage front end followed by angular dictionary matching and a compact 1D CNN can improve two-source grid-based DOA resolution under the primary low SNR, limited snapshot ULA profile. The method is evaluated against classical beamforming and subspace estimators, DA-MUSIC, SBL, off-grid SBL, and TS-MUSIC using the same snapshot matrices and deterministic trial seeds.

The main contributions of this work are as follows.

1. We propose an end-to-end framework integrating covariance matrix optimization and deep neural estimation to improve the direction-of-arrival estimation performance of two sources under low signal-to-noise ratio and limited snapshot conditions.
2. The covariance-processing front end and overcomplete angle dictionary map the covariance estimates onto physically interpretable real-valued directional response sequences.
3. A compact one-dimensional convolutional neural network without pooling layers regresses to generate grid-aligned pseudo-spectra, and peak discrimination is accomplished at its learning back end, without eigenvalue decomposition or iterative sparse optimization.

The remainder of this paper is organized as follows. Section 2 presents the array signal model and problem formulation. Section 3 describes the proposed estimation algorithm. Section 4 reports the simulation settings, evaluation metrics, and comparative analyses against representative baselines. Section 5 concludes the paper and discusses future work.

Unless otherwise stated, scalar variables are denoted by italic letters, e.g., N and λ; column vectors are denoted by bold lowercase letters, e.g., a and x; and matrices are denoted by bold uppercase letters, e.g., R and A. The superscripts ${\left(\cdot \right)}^{T}$, ${\left(\cdot \right)}^{H}$, and ${\left(\cdot \right)}^{-1}$ denote transpose, conjugate transpose, and inverse, respectively. The operator E[⋅] denotes the statistical expectation; tr(⋅) denotes the trace; and $∥\cdot {∥}_{F}$ denotes the Frobenius norm, where $∥A{∥}_{F}=\sqrt{tr\left(AA^{H}\right)}$. The operator vec(⋅) stacks a matrix columnwise into a vector; $I_{M}$ is the M×M identity matrix; and $C^{M\times N}$ and $R^{M\times N}$ represent complex and real matrix spaces of size M×N, respectively.

## 2. Signal Model

**Figure 1:**
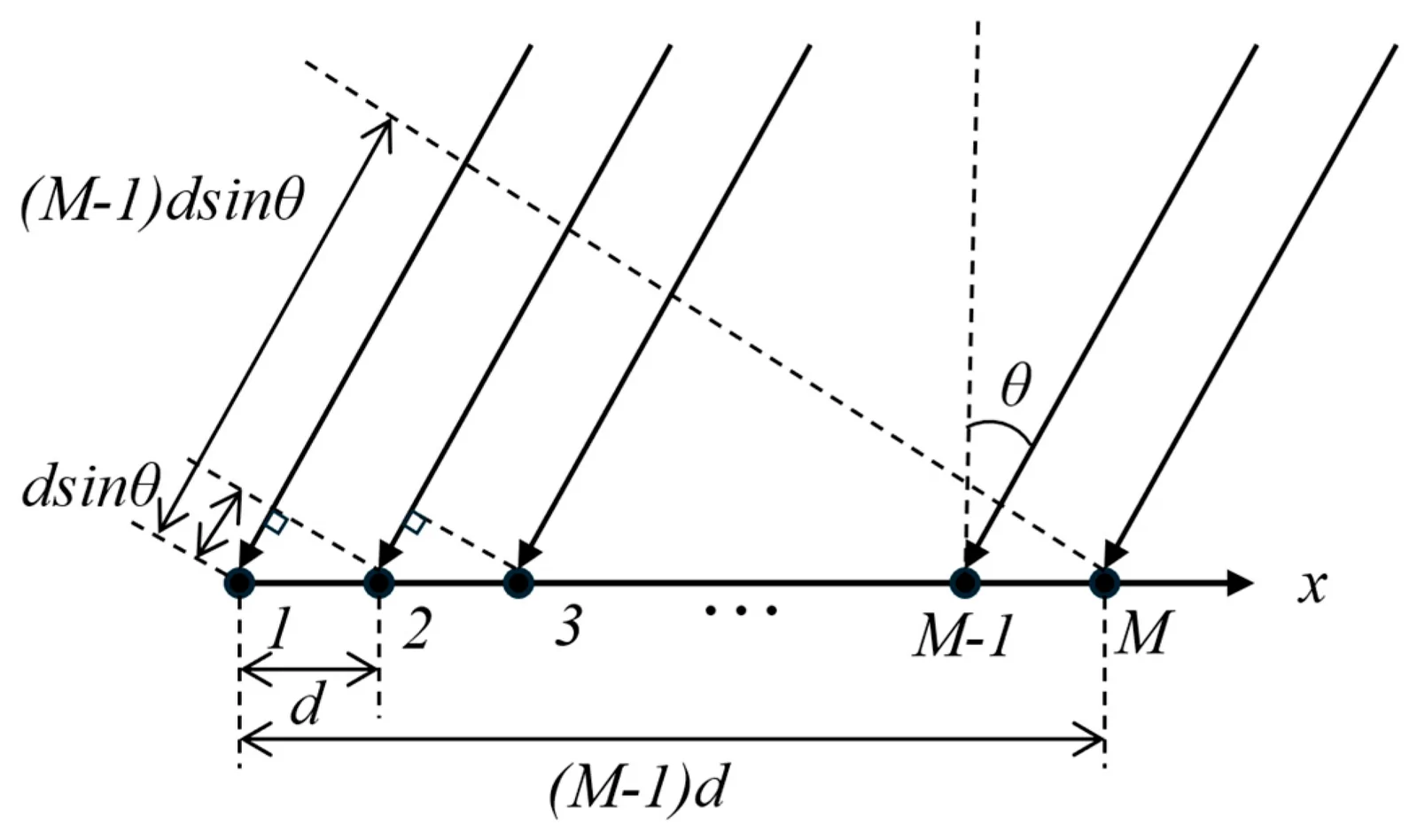
Uniform linear array model.

Consider a ULA consisting of M isotropic array elements. The interelement spacing is fixed at one half of the carrier wavelength, i.e., d=λ/2. Assume that K mutually uncorrelated far-field narrowband sources impinge on the array from distinct azimuth angles $\theta ={\left[\theta_{1},\theta_{2},\ldots ,\theta_{K}\right]}^{T}$, with K<M, as shown in Figure 1.At the tth snapshot, the received complex baseband vector is modeled as

(1) y(t)=A(θ)s(t)+n(t), t=1,2,…,N

Here, $A\left(\theta \right)=\left[a\left(\theta_{1}\right),a\left(\theta_{2}\right),\ldots ,a\left(\theta_{K}\right)\right]\in C^{M\times K}$ is the array manifold matrix. The steering vector associated with the kth source is $a\left(\theta_{k}\right)\in C^{M\times 1}$ and is given by

(2) $$a\left(\theta_{k}\right)={\left[1,e^{j\pi sin\theta_{k}},\ldots ,e^{j\left(M-1\right)\pi sin\theta_{k}}\right]}^{T}$$

In (2), $j=\sqrt{-1}$ is the imaginary unit. The vector $s\left(t\right)\in C^{K\times 1}$ contains the source signals at snapshot t, $n\left(t\right)\in C^{M\times 1}$ denotes additive environmental receiver noise, and N is the total number of effective snapshots within the coherent processing interval.

The K source sequences are generated independently as zero-mean unit-variance circular complex Gaussian samples. With steering matrix A(θ), the clean array data are X(t)=A(θ)s(t). The scene-specific clean-signal power is $Px=||X||F^{2}/\left(MN\right)$, and the reported SNR is defined as 10log10(Px/Pn), where Pn is the per-sensor noise variance. Spatially and temporally independent circular complex Gaussian noise with variance Pn is then added to X. Thus, the SNR is an array-output, scene-level power ratio rather than a per-source SNR. Equal source powers are used unless a different condition is explicitly stated.

Under the standard array signal processing assumptions, all source signals are zero-mean, stationary random processes and are mutually uncorrelated. Thus, the source covariance $R_{s}=E\left[s\left(t\right)s^{H}\left(t\right)\right]$ is diagonal and of full rank. The receiver noise follows a spatially independent and identically distributed zero-mean complex Gaussian white noise model, $n\left(t\right)∼CN\left(0,\sigma_{n}^{2}I_{M}\right)$, where $\sigma_{n}^{2}$ is the noise variance. The source and noise processes are statistically independent.

The population covariance matrix of the received vector is therefore

(3) $$\Sigma =E\left[y\left(t\right)y^{H}\left(t\right)\right]=A\left(\theta \right)R_{s}A^{H}\left(\theta \right)+\sigma_{n}^{2}I_{M}$$

In practice, the infinite time expectation in (3) is unavailable, and the algorithms must rely on a finite number of snapshots. The SCM is constructed as

(4) $$\hat{R}=\frac{1}{N}\sum_{t=1}^{N}y\left(t\right)y^{H}\left(t\right)$$

Classical model-driven algorithms, such as MUSIC and TLS-ESPRIT, rely on the orthogonality between the signal and noise subspaces obtained from the eigen decomposition of $\hat{R}$. When the SNR is high, and N≫M, the law of large numbers implies that $\hat{R}$ converges in probability to Σ. However, random matrix theory indicates that when the ratio between N and M approaches a finite constant, or when N<M and rank deficiency occurs, the statistical behavior of the SCM can deteriorate systematically. In multivariate statistics, if the observation vectors follow a complex Gaussian distribution, the unnormalized covariance $N\hat{R}$ follows a complex Wishart distribution with N degrees of freedom, denoted by ${CW}_{M}\left(N,\Sigma \right)$ [30].

In an extremely small snapshot regime, the eigenvalue distribution of $\hat{R}$ suffers from severe eigenvalue spread. Large eigenvalues are overestimated, while small eigenvalues shrink drastically and may even become zero. As a result, the extracted noise subspace of $\hat{R}$ contains considerable projection energy originating from the signal subspace, which violates the fundamental orthogonality condition $a^{H}\left(\theta \right)U_{n}=0$.

**Figure 2:**
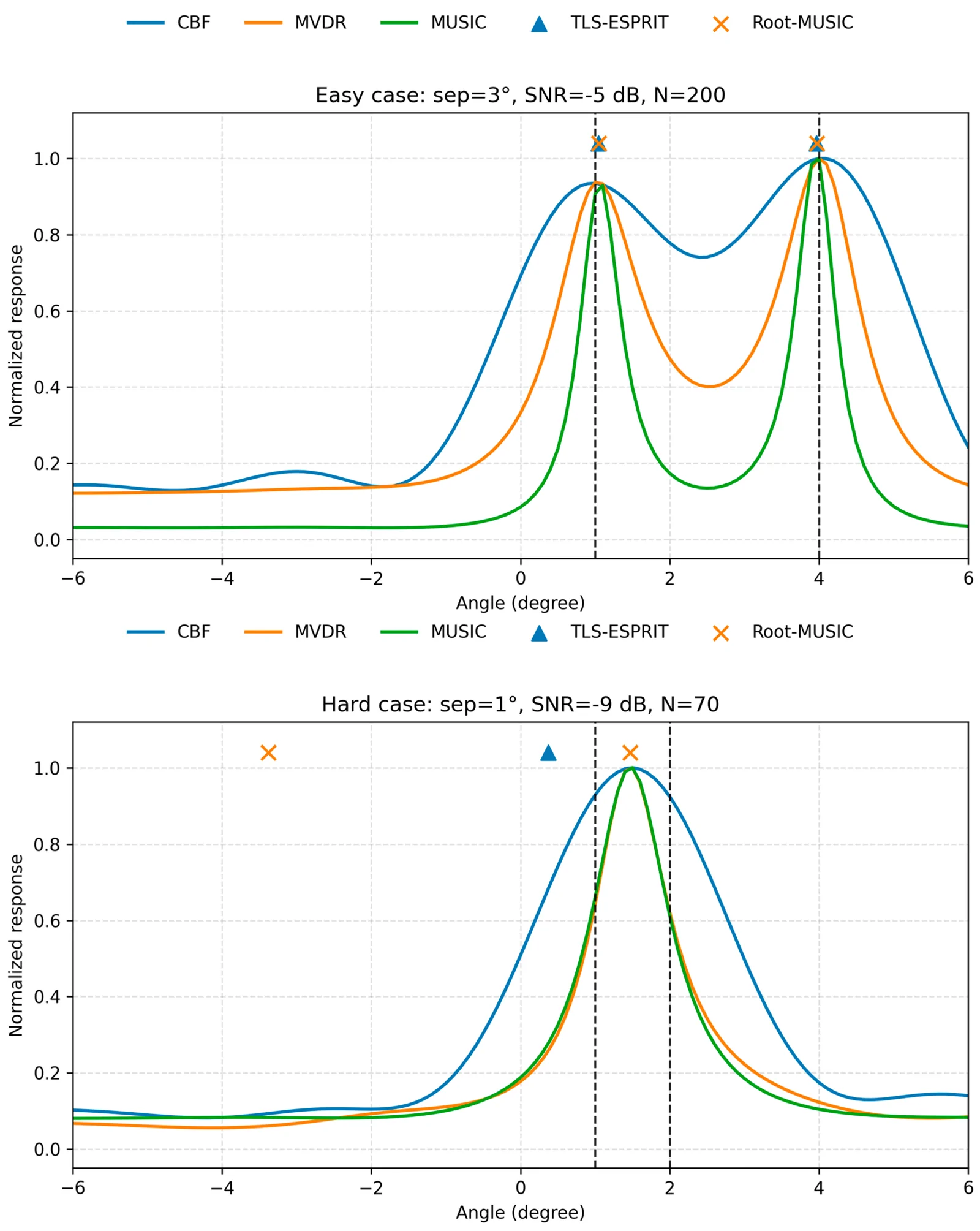
Impact of low SNR and limited snapshots on DOA estimation.

Figure 2 compares the spatial spectra of five DOA estimators. For a 3° separation, SNR = −5 dB, and *N* = 200, all five methods resolve the true angles (1° and 4°). For a 1° separation, SNR = −9 dB, and N = 70, the spectra show broadened lobes, shifted peaks, and fused responses, particularly for CBF and MVDR. These effects are consistent with finite-sample covariance error and subspace leakage and motivate the structured covariance regularization and angular-domain feature construction introduced in Section 3.

## 3. Toeplitz-Enhanced Neural Network for DOA Estimation

### 3.1. Overall Conceptual Architecture

**Figure 3:**
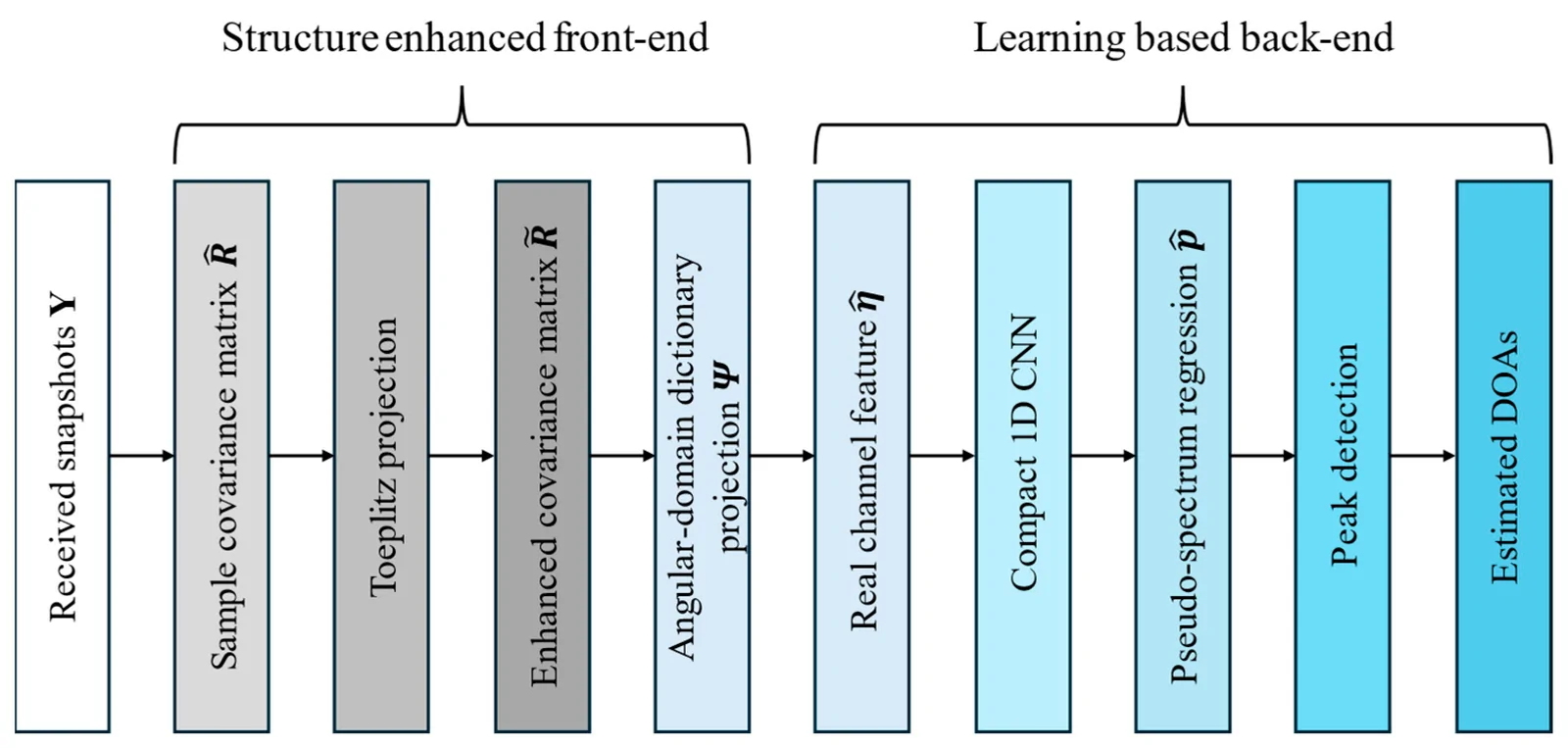
Workflow of the proposed TENN-DOA algorithm.

To address these challenges, TENN-DOA cascades covariance regularization, angular-domain feature projection, and grid-based spectrum regression. Under low SNRs and limited snapshots, the major difficulty in DOA estimation is not merely whether a neural network is sufficiently complex; rather, the input SCM itself has already been strongly perturbed. If the degraded covariance matrix is fed directly into the network, the network must simultaneously learn denoising, structural correction, and directional discrimination, which increases the training difficulty and weakens generalization under adverse conditions. TENN first enhances the sample covariance analytically by exploiting the physical structure of the ULA, then maps the result onto the angular feature domain, and finally estimates a discrete pseudo spatial spectrum through a lightweight convolutional network, the flow chart is shown in Figure 3.

TENN-DOA consists of a structure-enhanced front end followed by a learned spectrum regressor. The front end performs Hermitian–Toeplitz projection, data-adaptive linear shrinkage, and positive-semidefinite (PSD) eigenvalue clipping before matching the resulting covariance to an angular dictionary. The back end maps the real, grid-aligned response sequence to a discrete pseudo-spectrum with a compact 1D CNN, after which the local peak selection returns the DOA estimates.

The proposed method can be summarized in four steps:

1. Construct the SCM from the array snapshot data;
2. Project the SCM onto the Hermitian–Toeplitz set, fuse the target with the SCM by linear shrinkage, and project the fused matrix onto the PSD cone by eigenvalue clipping;
3. Construct the direction-related features with an overcomplete angular-domain dictionary and retain the real-valued response;
4. Regress a discrete pseudo-spectrum with a compact 1D CNN and obtain the grid-constrained DOA estimates by peak detection.

### 3.2. Covariance Enhancement Based on Toeplitz Structure

**Figure 4:**
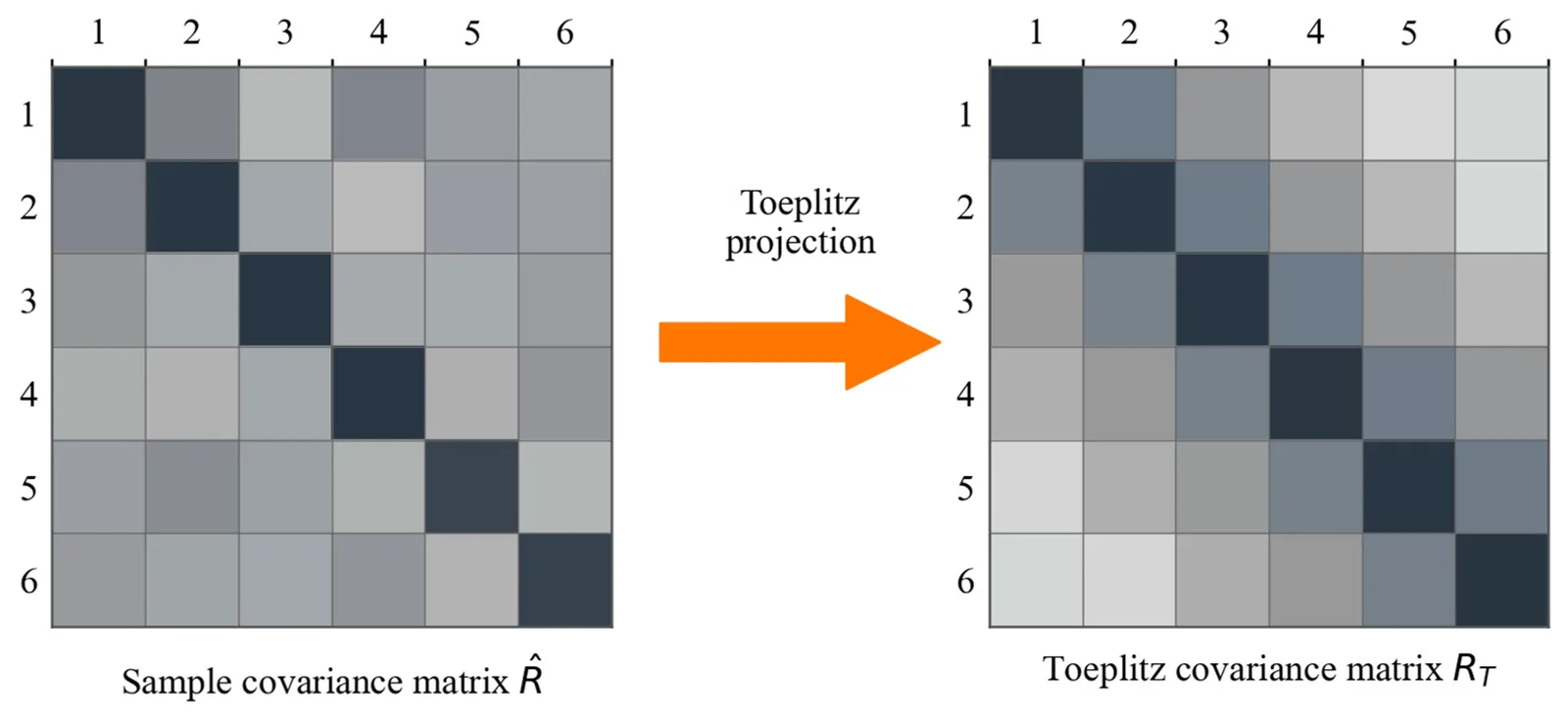
Illustration of Toeplitz projection.

To reduce finite snapshot fluctuations, the ULA covariance structure is used as a regularizing prior. For an ideal calibrated ULA with spatially stationary statistics, the population covariance is Hermitian–Toeplitz because the entries on a common diagonal share the same expectation, as shown in Figure 4.

The proposed method first projects the SCM onto this structure to obtain a Toeplitz target matrix $R_{T}$. Let m denote the diagonal offset. The projection is expressed as

(5) $$R_{T}=\sum_{m=-\left(M-1\right)}^{M-1}\frac{1}{M-\left|m\right|}tr\left(\hat{R}J_{m}\right)J_{-m}$$

Here, $J_{m}$ is a shift matrix. Averaging the entries on each diagonal is the orthogonal projection of the SCM onto the Hermitian–Toeplitz subspace. By pooling repeated estimates of the same covariance lag, this operation reduces the elementwise finite-sample fluctuations while preserving the ULA structural prior.

Under array mismatch or nonideal disturbances, a strict Toeplitz constraint can introduce bias. We therefore balance the sample information and the structured target through linear shrinkage. The enhanced covariance is formed as the convex combination in (6):

(6) $$\tilde{R}=\left(1-\alpha \right)\hat{R}+\alpha R_{T}$$

In (6), α is an estimated shrinkage intensity adapted from the Toeplitz target covariance estimator in [30]. The underlying criterion is the expected squared Frobenius risk $E||\tilde{R}\left(\alpha \right)-R|{|}_{F}^{2}$ under complex Gaussian observations, for which the unnormalized SCM follows a complex Wishart law. Because the Toeplitz target is estimated from the same SCM, the closed-form coefficient is an estimator of the risk-minimizing intensity rather than a distribution-free optimum. The implementation clips α to [0, 1] and uses ε = 10^−12^; if the denominator in (7) is not larger than ε, α is set to 1.

(7) $$\alpha^{⋆}=\frac{\left(N-3\right)tr\left({\hat{R}}^{2}\right)+\left(N-1\right){tr}^{2}\left(\hat{R}\right)}{\left(N-2\right)\left(N+1\right)tr\left[{\left(\hat{R}-R_{T}\right)}^{2}\right]}$$

To ensure that the coefficient remains in the valid convex interval, the implemented value is clipped as

(8) $$\alpha =min\left\{max\left(\alpha^{⋆},0\right),1\right\}$$

When the SCM differs substantially from its Toeplitz projection, the denominator in (7) increases and the clipped coefficient tends to retain more sample information; when the discrepancy is small, the estimate approaches the Toeplitz target. The rule is model-dependent. In a 1000-trial diagnostic with SNR = −5 dB and *N* = 100, the mean α changed from 0.9998 under complex Gaussian noise to 0.8235 for complex Student-t noise (3 degrees of freedom) and 0.2703 for 1% contaminated Gaussian noise with 20-fold impulses.

Diagonal averaging guarantees a Hermitian–Toeplitz target but not positive semidefiniteness. Accordingly, after shrinkage the implementation first symmetrizes the fused covariance, computes its Hermitian eigen decomposition, replaces every eigenvalue λi by max(λi, 0), and reconstructs the covariance before angular matching. In a 2000-trial audit of the M = 16 wide-angle profile, 34 matrices (1.7%) had a minimum eigenvalue below 10^−10^; the minimum was −0.679. The PSD projection changed those matrices by 0.475% on average in the relative Frobenius norm (maximum 2.824%), and did not change the selected DOA pair in the 34 affected cases.

### 3.3. Angular-Domain Dictionary Projection and Feature Construction

After obtaining the enhanced covariance matrix $\tilde{R}$, directly treating it as an M×M complex image and feeding it into a CNN would introduce $O\left(M^{2}\right)$-dimensional redundancy and would ignore the fact that DOA estimation is essentially a one-dimensional spatial manifold inference problem. Therefore, an overcomplete angular dictionary is introduced to project and reduce the dimensionality of the covariance representation.

Let the search range be $\phi \in \left[\phi_{min},\phi_{max}\right]$. With a high-resolution angular step Δϕ, the range is divided into L grid points $\Phi =\{\phi_{1},\phi_{2},\ldots ,\phi_{L}\}$. For each candidate angle $\phi_{l}$, an ideal noise-free steering vector $a\left(\phi_{l}\right)$ is generated, and a rank-one covariance atom is defined as $B\left(\phi_{l}\right)=a\left(\phi_{l}\right)a^{H}\left(\phi_{l}\right)$. Vectorizing all atoms columnwise gives the overcomplete feature-mapping dictionary:

(9) $$\Psi =\left[vec\left(B\left(\phi_{1}\right)\right),vec\left(B\left(\phi_{2}\right)\right),\ldots ,vec\left(B\left(\phi_{L}\right)\right)\right]\in C^{M^{2}\times L}$$

The enhanced covariance matrix is vectorized and matched to the angular dictionary to obtain a one-dimensional direction response sequence:

(10) $$\hat{\eta }=\Psi^{H}vec\left(\tilde{R}\right)$$

**Figure 5:**
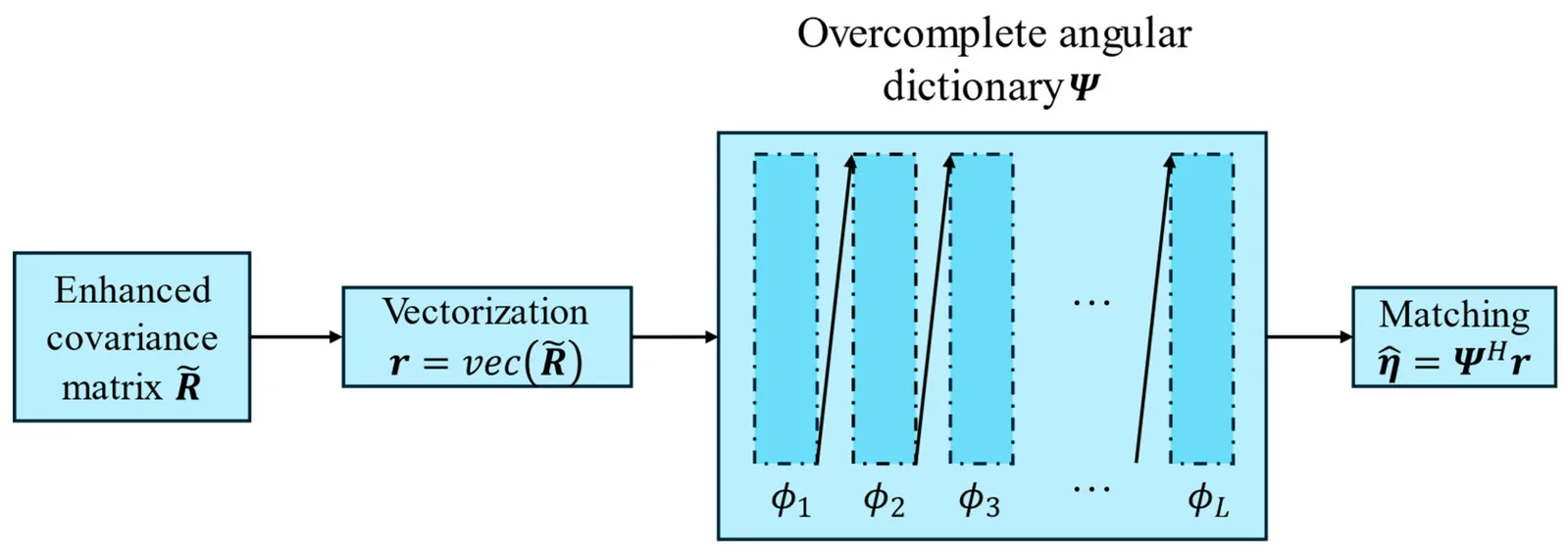
Angular-domain dictionary projection and real-channel feature construction.

As illustrated in Figure 5, the lth response equals $vec{\left(B\left(\varphi_{l}\right)\right)}^{H}vec\left(\tilde{R}\right)=a{\left(\varphi_{l}\right)}^{H}\tilde{R}a\left(\varphi_{l}\right)$. Because $\tilde{R}$ is Hermitian, this quadratic form is real. The model is therefore interpreted as effectively real valued.

(11) $$X_{in}=Re\left(\eta \right)\in R^{L}$$

### 3.4. Network Architecture

**Figure 6:**
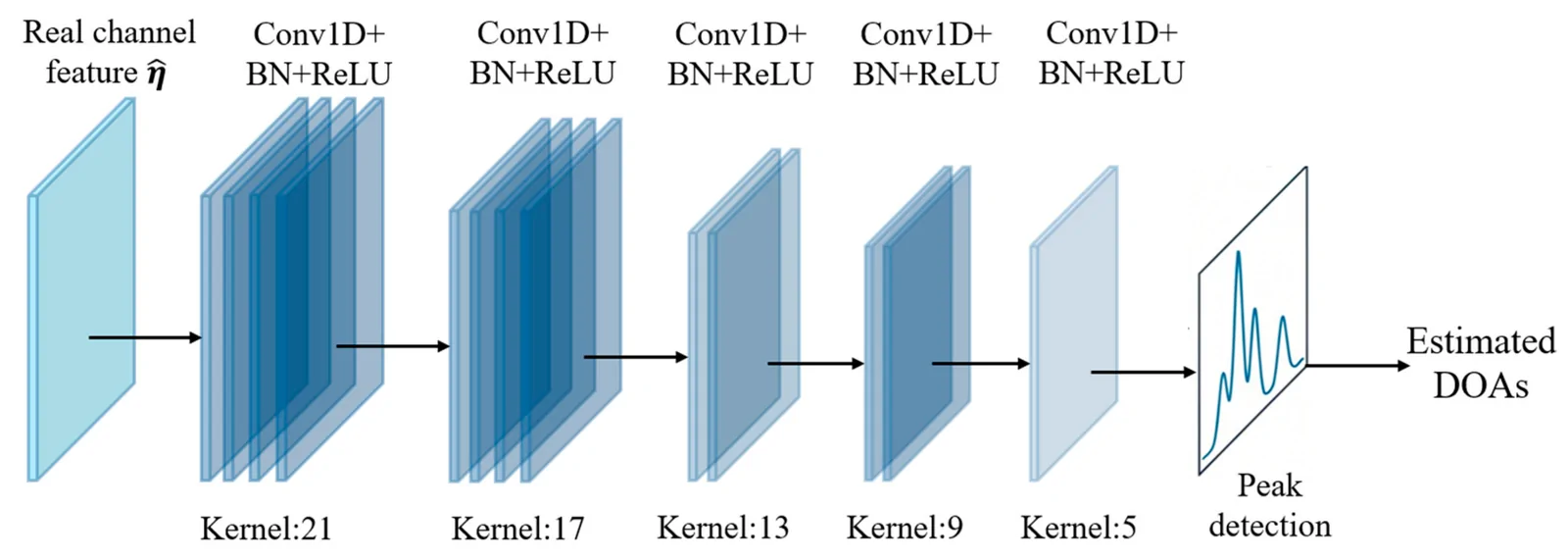
Architecture of the lightweight 1D CNN.

The back end regresses a discrete pseudo-spectrum from the real, grid-aligned response sequence. No pooling is used; every convolution has stride 1 and symmetric zero padding, so the output length remains aligned with the angular grid. The primary comparison model uses kernel sizes 21, 15, 11, 5, and 3 (effective receptive field: 51 bins). The controlled ablation uses 21, 17, 13, 9, and 5 (61 bins) versus a 3, 1, 1, 1, 1 small-kernel stack. The hidden layers use batch normalization and ReLU, and the final convolution is followed by a sigmoid in the trained profiles. These empirical schedules are evaluated in the ablation study, as shown in Figure 6.

Grid mismatch produces a local leakage pattern across neighboring dictionary points. The larger early kernels aggregate this local envelope, while later smaller kernels refine the predicted peak. This mechanism can improve the robustness to off-grid inputs, but peak-to-grid mapping remains quantized.

For the main narrow-angle profile, the network is trained by minimizing the MSE between the sigmoid output and a two-hot label. The label contains two positive bins among 121 positions; no explicit class reweighting is used, and the number of sources K = 2 is assumed known. The wide-angle grid-pair profiles use binary cross entropy. Model selection is based on the lowest validation loss.

(12) $$L=\frac{1}{L}\sum_{l=1}^{L}{\left({\hat{p}}_{l}-z_{l}\right)}^{2}$$

After inference, local maxima are detected with a minimum separation of five grid bins. The K = 2 highest local peaks are selected; if fewer than two local maxima are found, the two largest output bins are used as a deterministic fallback. The default estimator maps these bins to the grid angles and is grid-constrained.

## 4. Simulation Analysis and Discussion

### 4.1. Simulation Setup

Two different simulation configuration files are adopted, as shown in Table 2. The primary resolution and ablation profile uses *M* = 40, *K* = 2, a [−6°, 6°] grid with 0.1° spacing (*L* = 121), and 43,200 generated scenes. The dataset is split deterministically into 34,560 training and 8640 validation scenes using seed 42; the test scenes are generated independently and never drawn from this pool. The training SNR is sampled uniformly from [−10, 10] dB and *N* = 70. The wide-angle scans use separate *M* = 16 models: [−60°, 60°] and [−90°, 90°] grids at 1° spacing, with 36,300 and 81,450 grid-pair scenes, respectively; SNR values of {−20, −15, −10, −5,0} dB; and snapshot choices of {100, 200, 500, 1000}. Consequently, results outside a profile’s training support are identified as generalization tests, and a [−60°, 60°] model is never used to produce [−90°, 90°] outputs. Adam uses a learning rate of 10^−3^ and batch size of 64. The main profile is trained for up to 300 epochs; the controlled ablation uses 300 epochs.

TS-MUSIC applies the same Toeplitz projection, shrinkage coefficient, and PSD projection used by TENN-DOA, followed by a conventional MUSIC eigenspace and pseudo-spectrum calculation; it contains no CNN. This structured baseline isolates the effect of the learned spectrum regressor from the covariance front end.

The RMSE is computed after the minimum squared error assignment between the two estimates and two true angles. The PoR declares a trial resolved when both matched absolute errors are smaller than half the true separation. Because *K* = 2 is fixed and the fallback always returns two estimates, false positives and false negatives are not reported as separate source count metrics; missed or spurious peaks appear as large, matched errors and failed PoR trials.

(13) $$RMSE=\sqrt{\frac{1}{CK}\sum_{c=1}^{C}\sum_{k=1}^{K}{\left({\hat{\theta }}_{k,c}-\theta_{k}\right)}^{2}}$$

Here, C is the number of Monte Carlo trials, K is the number of sources, and ${\hat{\theta }}_{k,c}$ is the estimated angle of the kth target in the cth trial.

The PoR measures the ability to distinguish adjacent sources. Let the true angular separation be $\Delta \theta =\left|\theta_{1}-\theta_{2}\right|$. A trial is regarded as successful if all optimally matched estimation errors satisfy

(14) $$max\left(\left|{\hat{\theta }}_{1}-\theta_{1}\right|,\left|{\hat{\theta }}_{2}-\theta_{2}\right|\right)<\frac{\Delta \theta }{2}$$

The PoR is the fraction of resolved trials. The main comparison uses 10 independent repeats of 50 test scenes per operating point (500 scenes), with the deterministic Seed Sequence derived seeds from base seed 42, as shown in Table 3. The ablation fixed point and each ablation SNR point use 300 independent scenes with test seed base of 880,000. The RMSE uncertainty is summarized by a 2000-resample scene-level bootstrap 95% interval; the PoR uses a Wilson 95% interval.

### 4.2. Resolution and Accuracy Performance Evaluation

#### 4.2.1. Resolution Performance Under Different SNRs

**Figure 7:**
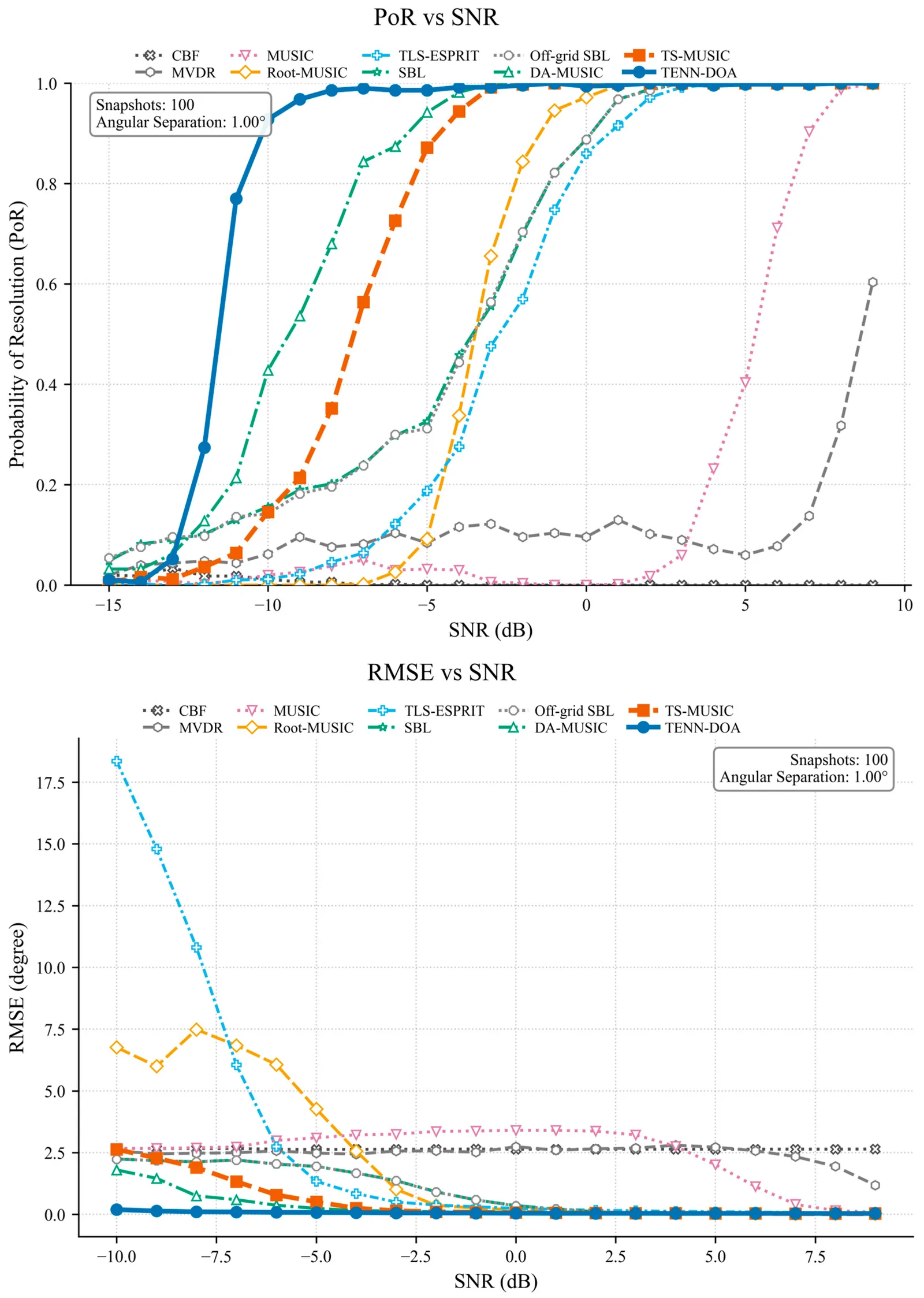
PoR and RMSE comparison under different SNRs.

To analyze noise resistance, the two sources were fixed in a closely spaced configuration with an angular separation of approximately $1^{\circ }$, and the number of snapshots was set to N=100. The SNR was swept from −15 dB to 10 dB. The PoR and RMSE trends of all algorithms are shown in Figure 7.

The results show the low SNR degradation of the eigenspace-based methods. DA-MUSIC improves over several classical methods at a moderate negative SNR, while its performance also decreases at the lowest SNR values. Within the same simulated profile, TENN-DOA benefits from the structured covariance estimate and the learned, grid-aligned response mapping.

At SNR = −10 dB and *N* = 100, TENN-DOA therefore maintains a resolved trial probability close to 0.94 for the 1° pair, while most non-learned baselines have a PoR below 0.15, except DA-MUSIC. At 0 dB, DA-MUSIC and TS-MUSIC also reach a PoR of 1.000, so the advantage is concentrated in the low SNR portion of this simulation.

#### 4.2.2. Resolution Performance for Closely Spaced Targets

**Figure 8:**
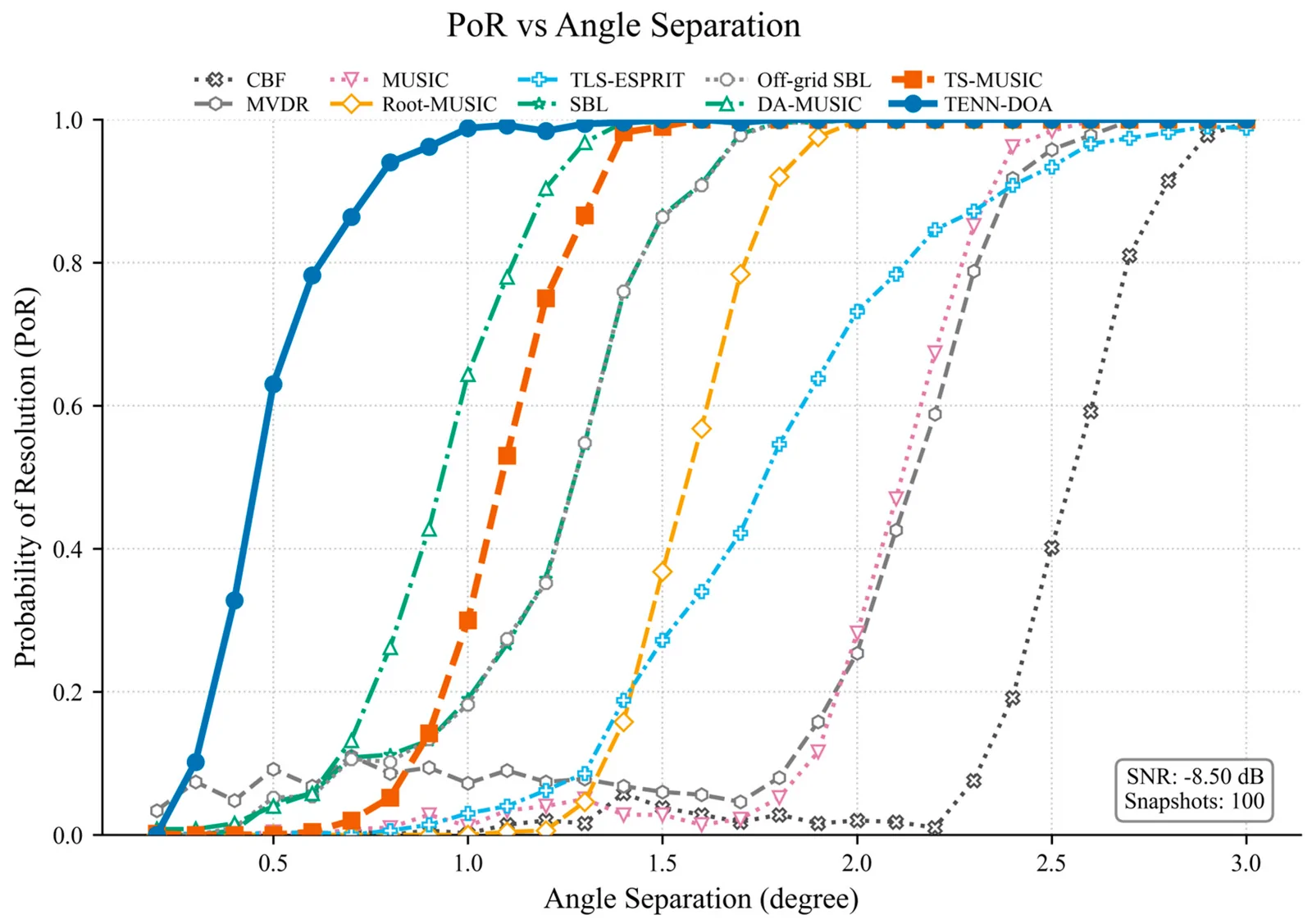

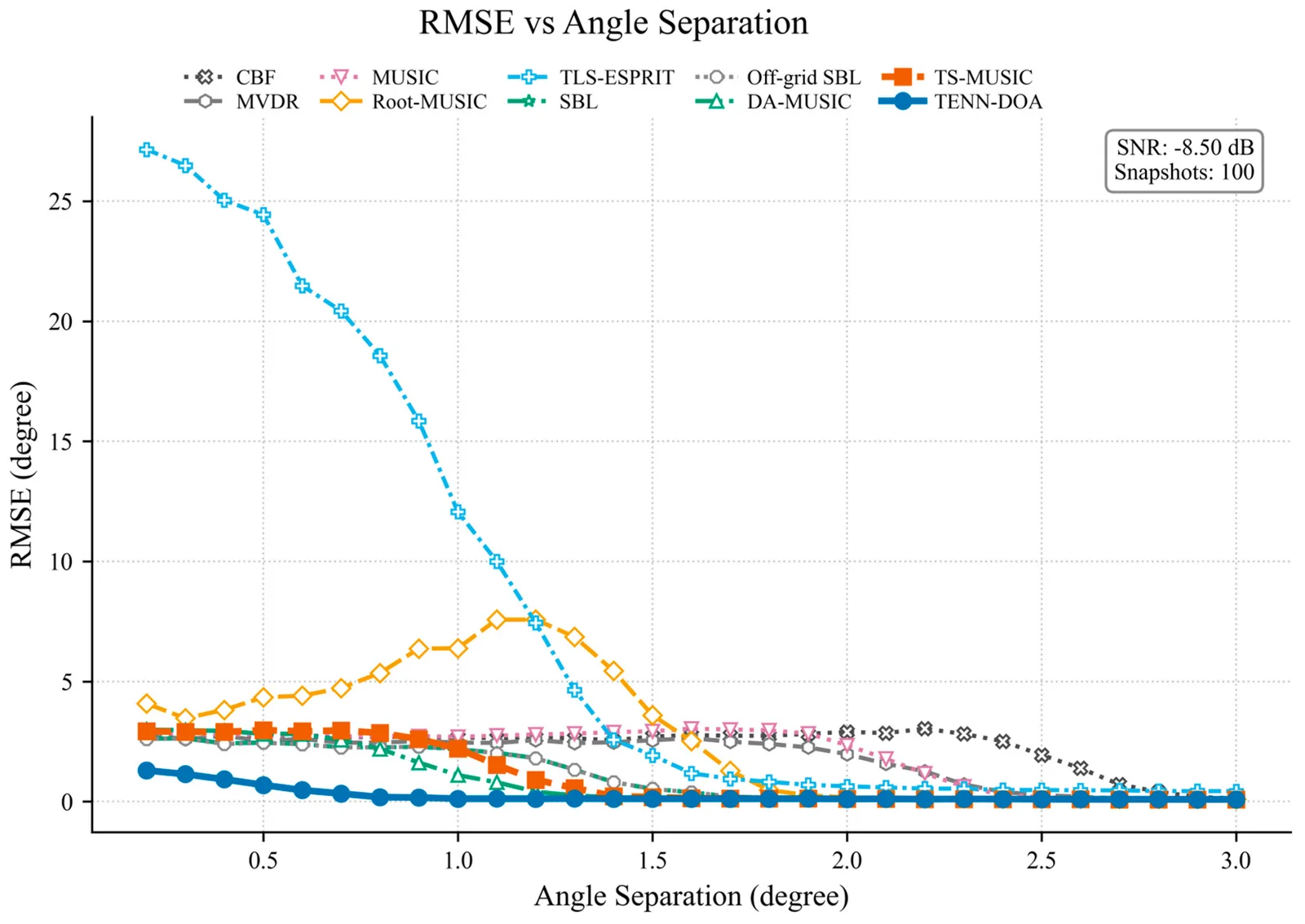
PoR and RMSE comparison under different angular separations.

To evaluate adjacent-peak separation, the SNR is fixed at −8.5 dB and the number of snapshots at *N* = 100. The angular separation is swept from 0.2° to 3.2°, and the resulting PoR and RMSE values are shown in Figure 8.

The results show that the PoR of TENN-DOA rises as the separation increases and exceeds 90% at Δ*θ* = 0.8°. Most conventional methods reach a comparably stable region only at larger separations, and the TENN-DOA RMSE is lower at the smallest tested separations. DA-MUSIC approaches the proposed method as the separation increases. These results support the improved adjacent-peak discrimination of TENN-DOA within this specific SNR, snapshot, array, and two-source profile.

#### 4.2.3. Snapshot Utilization Efficiency

The number of snapshots is a critical factor for covariance-based DOA estimators. With few snapshots, the covariance estimation error increases substantially and degrades the peak localization and target resolution.

**Figure 9:**
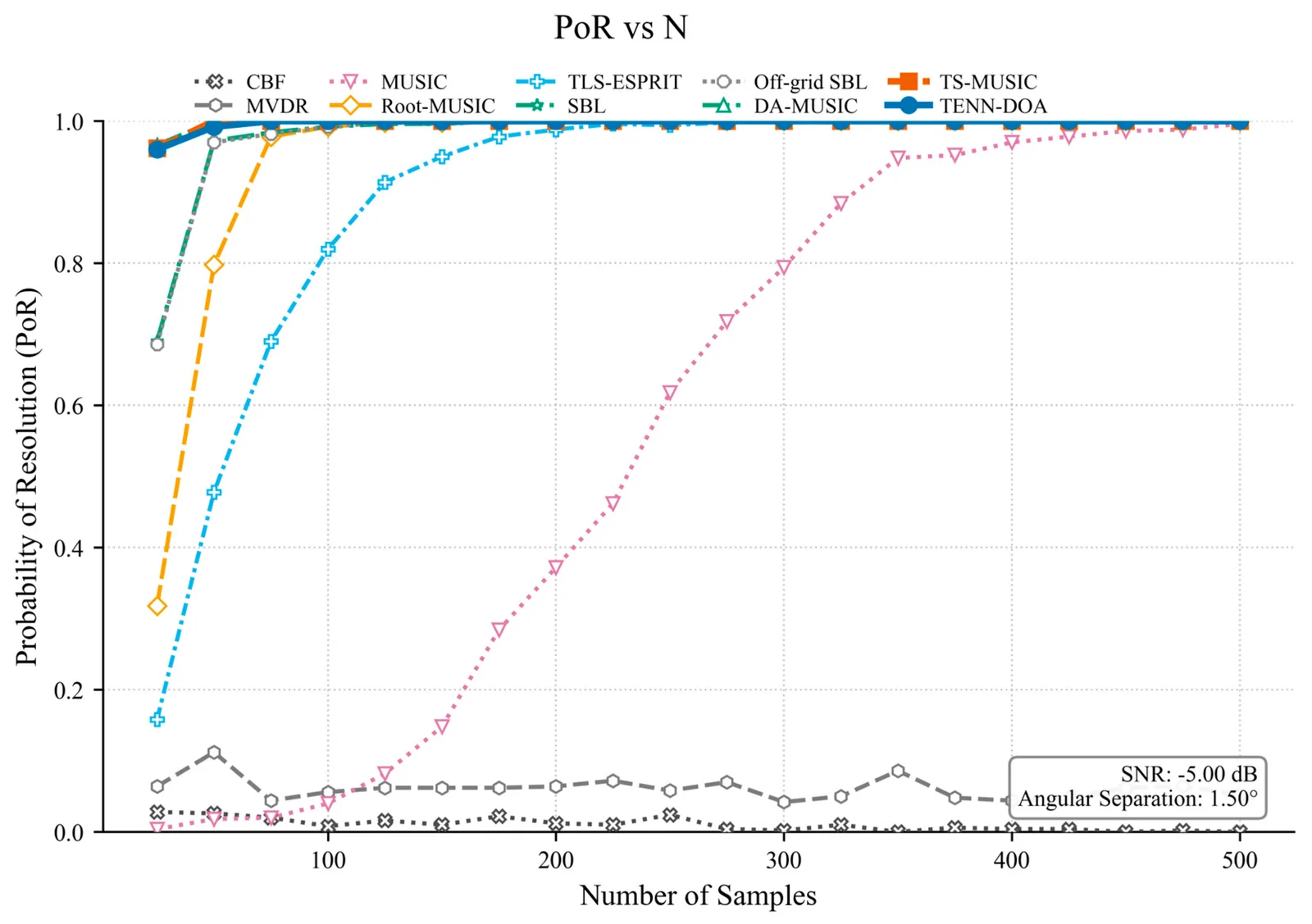

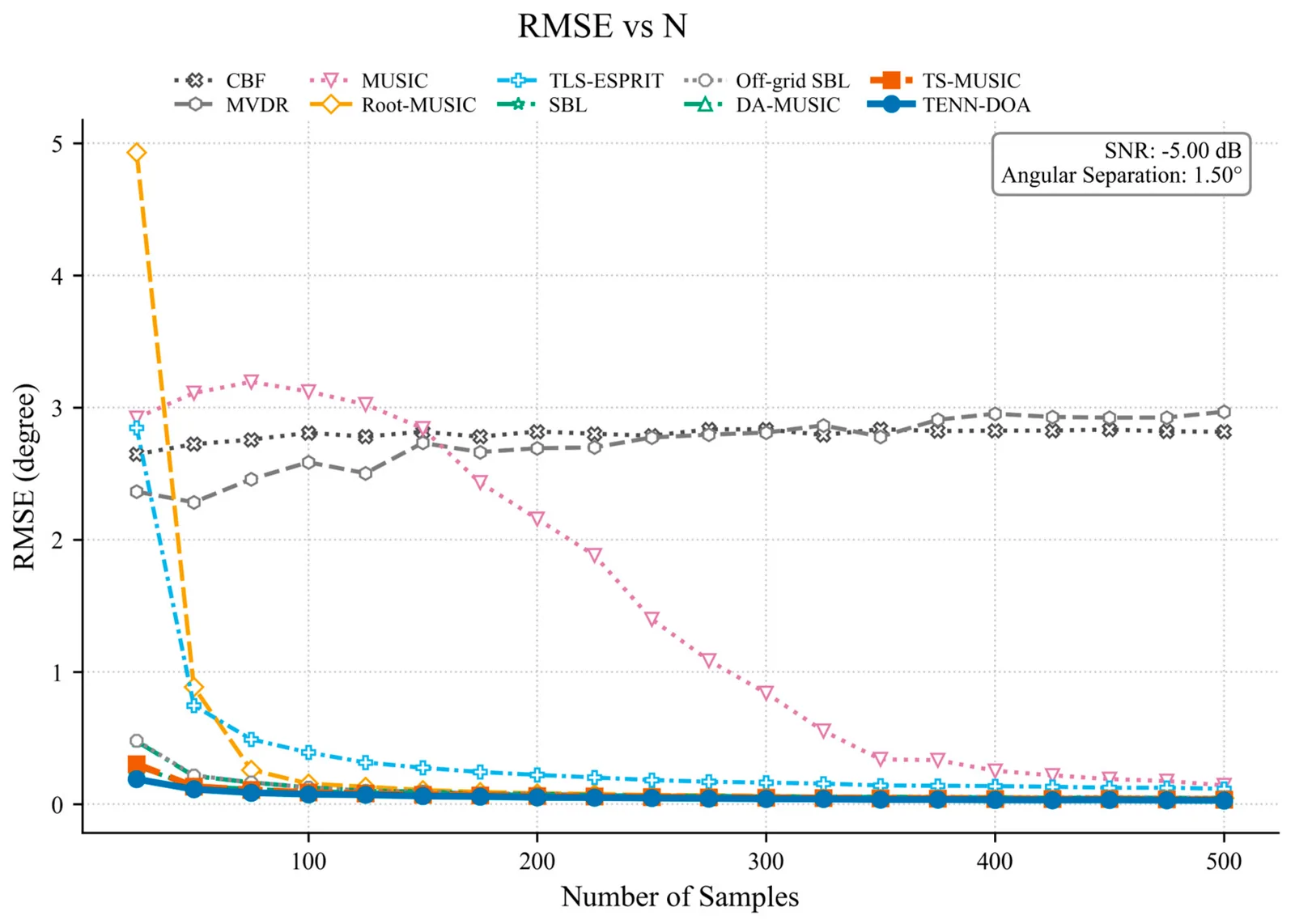
PoR and RMSE comparison under different numbers of snapshots.

For the snapshot-count sweep, the two sources are separated by approximately 1.5°, the SNR is fixed at −5 dB, and the number of snapshots increases from 25 to 500. Figure 9 reports the corresponding PoR and RMSE for each method.

At the tested −5 dB and approximately 1.5° separation, TENN-DOA attains a PoR above 90% at *N* = 25, whereas the conventional methods require more snapshots to reach their higher PoR regimes. Root-MUSIC and TLS-ESPRIT improve as the number of snapshots increases. In the small-sample portion of this sweep, the structured front end and CNN yield lower error for TENN-DOA, while conventional and sparse estimators remain competitive in less adverse regimes.

#### 4.2.4. Section Summary

Taken together, the three separate sweeps show a favorable PoR and RMSE for TENN-DOA at the investigated low SNR, small separation, and limited snapshot operating points. This method is well suited for passive sensing tasks with limited observation time where it is difficult to accumulate a large volume of snapshot data.

### 4.3. Ablation Study

The ablation study uses a fixed adverse two-source scenario with true DOAs [2.5°, 3.5°] (separation 1°), *N* = 70, and SNR = −5 dB; the RMSE is additionally scanned from −10 to 10 dB. Each point uses 300 independent test scenes. This design isolates the covariance enhancement, angular matching, and receptive field choices while keeping the generated scenes and optimizer settings matched.

The ablation study considers three key modules:

1. The Toeplitz structural projection module, which tests the ability of covariance structural constraints to suppress small snapshot statistical fluctuations;
2. The angular-domain dictionary projection module, which tests the benefit of direction-related feature construction for pseudo-spectrum learning;
3. The progressive lightweight convolutional kernel design, which tests whether large kernels followed by layerwise refinement outperform simple small-kernel stacking.

Variant A1 removes both the covariance enhancement and angular matching and feeds the real parts of the raw vectorized SCM to a CNN. Variant A2 retains the Toeplitz projection and shrinkage but removes angular matching. Variant A3 retains the front end and replaces the 61-bin progressive receptive field with the 3-bin small-kernel stack. All variants use the same generated scenes, split, optimizer, and evaluation seeds.

The number of trainable parameters of the A0 convolutional neural network is 13,037. On an NVIDIA GeForce RTX 2080 Ti graphics card equipped with CUDA-supported PyTorch 2.13.0+cu130, when the training batch size is 64, the neural network inference alone takes 0.62 milliseconds. The total time consumption of dictionary projection + convolutional neural network + peak selection is 1.71 milliseconds. Under the same local benchmark test conditions, the MUSIC algorithm takes 0.97 milliseconds. The above-mentioned test results are averaged from multiple simulations.

**Figure 10:**
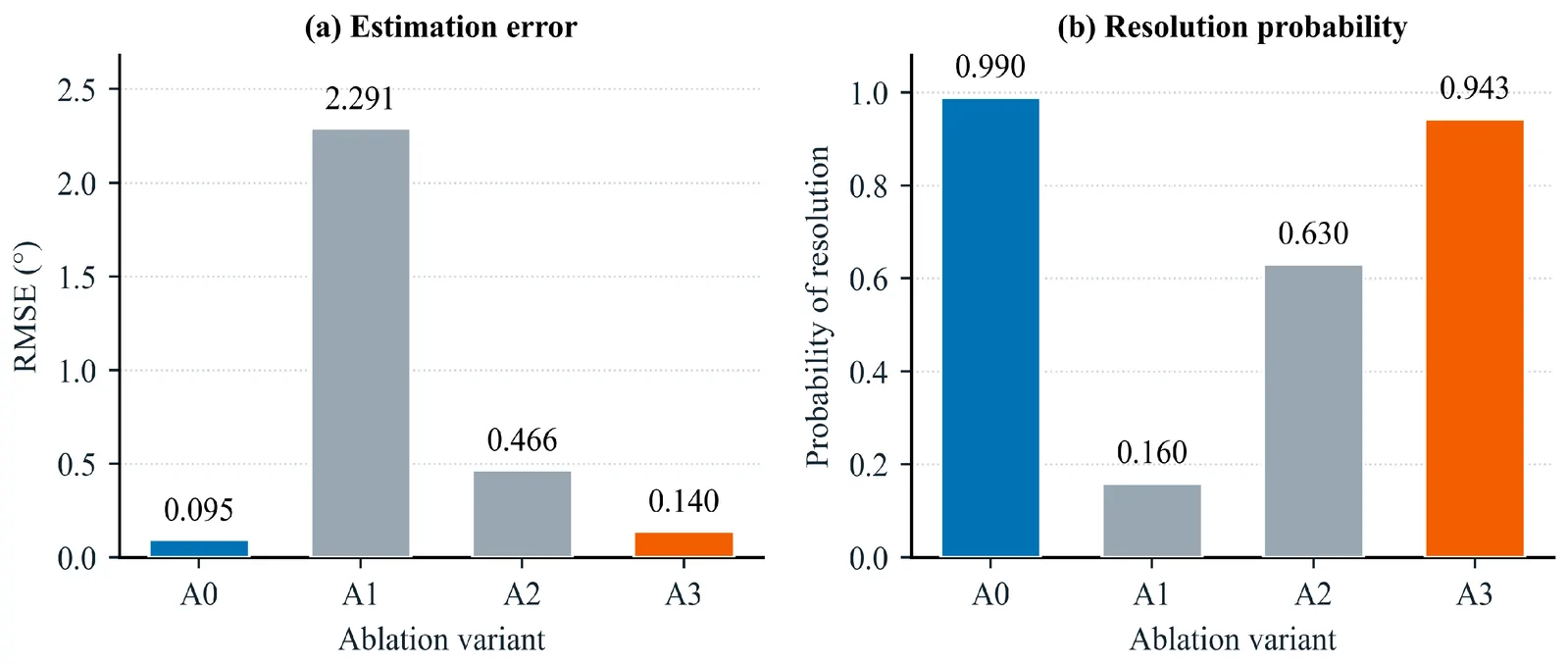
RMSE and PoR comparison for the ablation variants.

Figure 10 shows that A1 has a substantially larger error than the other variants and a PoR below 0.25 in the fixed adverse case. Thus, for the tested combination of low SNR, limited snapshots, and 1° separation, learning directly from the raw SCM does not reliably produce two resolved peaks. The comparison indicates that covariance enhancement and angular-domain matching account for much of the observed improvement in this controlled ablation.

A2 retains the Toeplitz projection and linear shrinkage but removes the angular-domain dictionary matching. It improves over A1 but remains below the full model in this ablation. This result is consistent with the dictionary transforming the covariance structure into a direction response sequence aligned with the angular grid, thereby reducing the burden on the CNN to infer the array geometry from the covariance elements.

A3 retains the complete front end but replaces the progressive large-to-small kernel schedule with a small-kernel stack. Its lower accuracy in this simulation indicates that the broader receptive field contributes to closely spaced peak discrimination.

**Figure 11:**
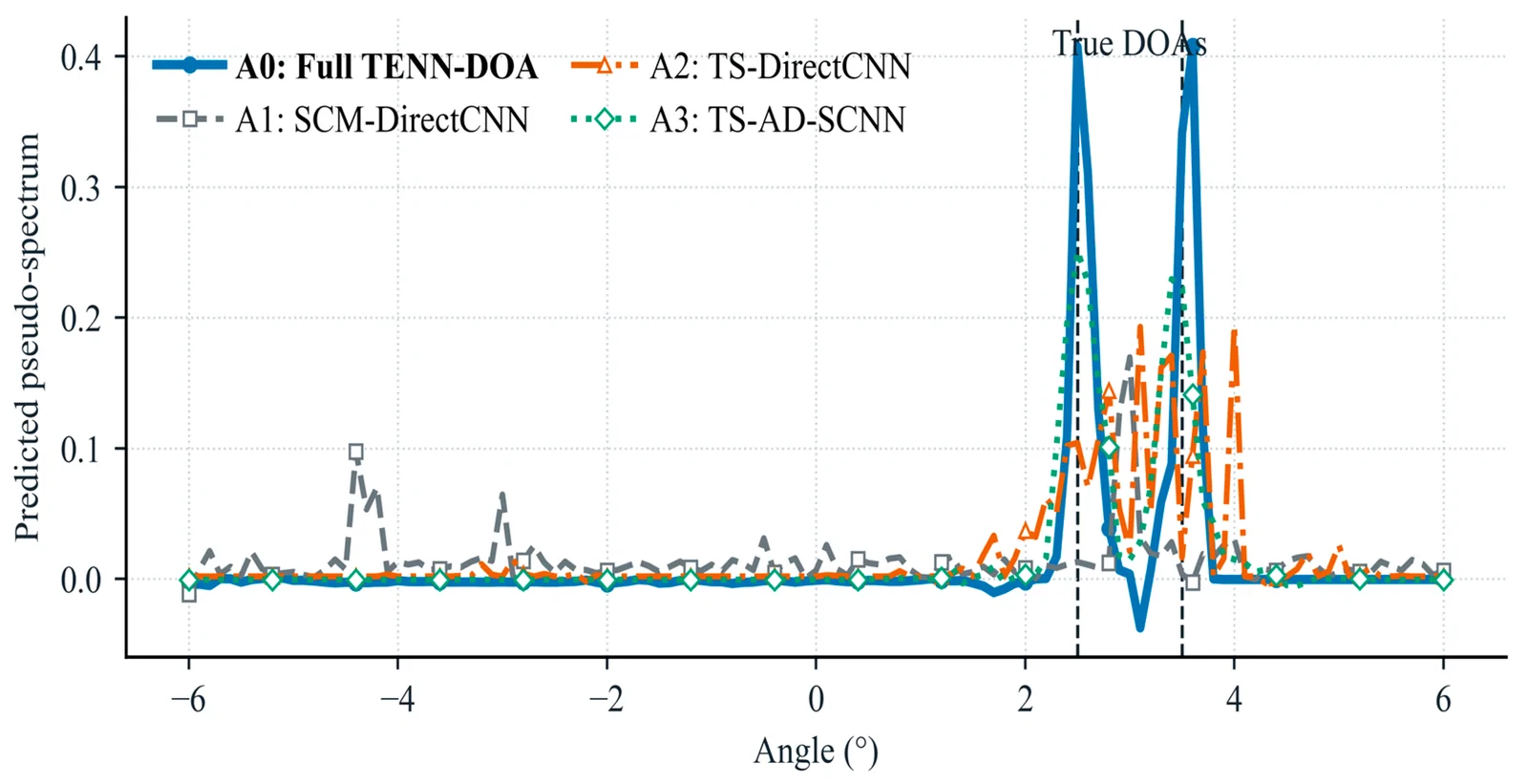
Pseudo spatial spectra of the ablation variants in the fixed adverse scenario.

Figure 11 explains the numerical differences at the spectral shape level. A1 uses the raw vectorized SCM without Toeplitz/shrinkage processing or angular matching, consistent with Table 4. A2 retains covariance enhancement but removes angular matching, whereas A3 retains the front end with the small-kernel back end.

**Figure 12:**
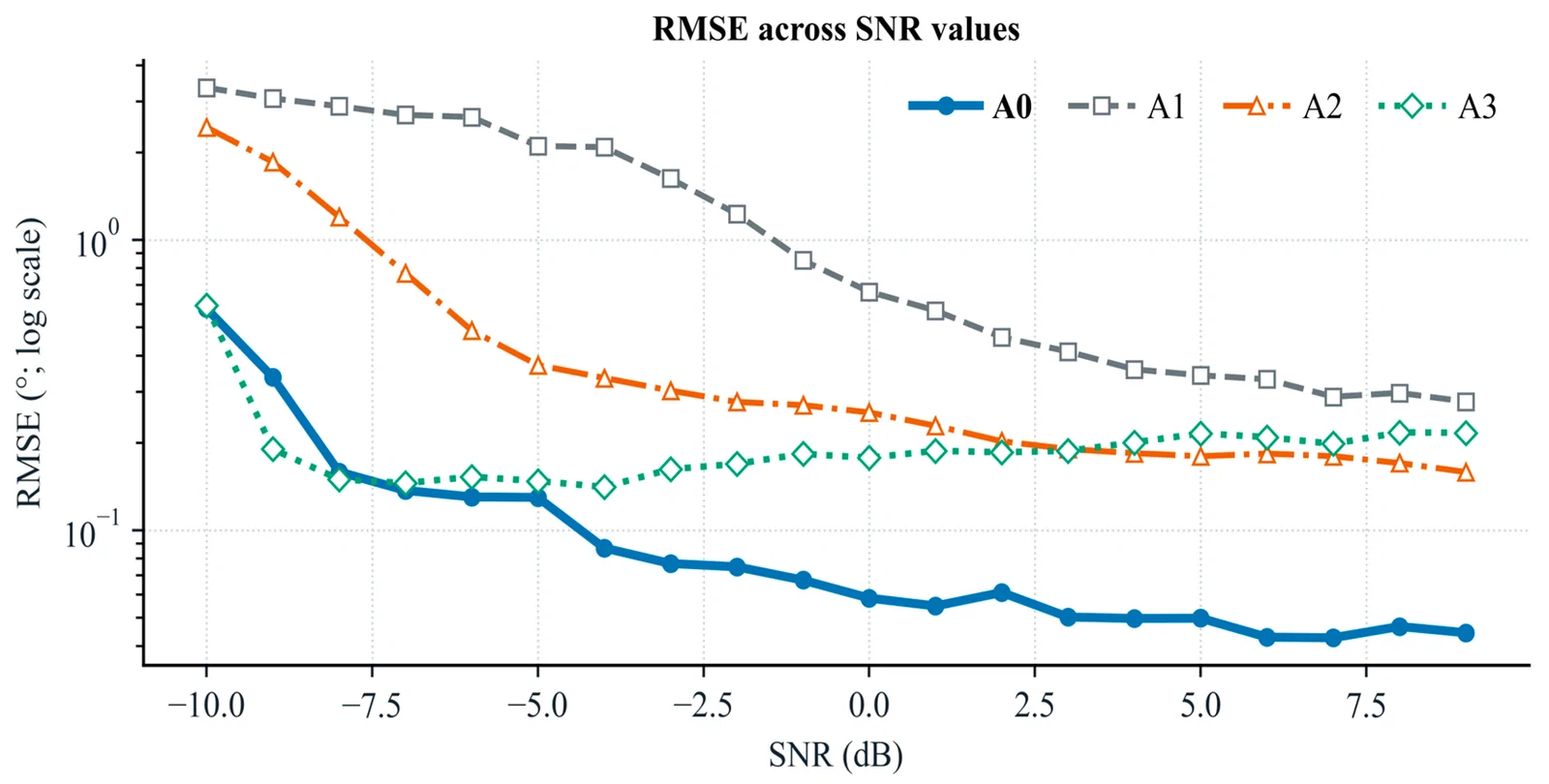
RMSE curves of the ablation variants under different SNRs.

Figure 12 shows the RMSE from −10 dB to 8 dB. At −10 dB, A0 and A3 have markedly lower RMSEs than A1 and A2. At 0 dB, A0 still achieves the lowest RMSE among the compared variants. This indicates that the benefits of structural enhancement and angular projection are not limited to a single test point; they persist over low-to-moderate SNRs.

### 4.4. Off-Grid DOA Estimation and Error Analysis

To further evaluate the generalization ability of TENN-DOA under nonintegral grid angles, three representative off-grid two-target scanning scenarios were analyzed. Each simulation used two sources and scanned the true DOA point by point over the search interval with a fixed angular separation. The estimated trajectories, per sample errors, and overall RMSE were recorded. Compared with tests at a few fixed angles, the scan-based simulations more fully reveal stability differences near the boundaries, end-fire directions, and closely spaced targets.

#### 4.4.1. Scenario 1: Low SNR with Moderate Angular Separation

In Scenario 1, the two targets are separated by $4.7^{\circ }$, the SNR is −10 dB, the first target is scanned with a fixed step over the specified angular domain, and the second target maintains the fixed separation from the first. The SCM is estimated to be 1000 snapshots. This scenario evaluates the tracking and estimation stability for off-grid two-target DOA estimation under a low SNR.

**Figure 13:**
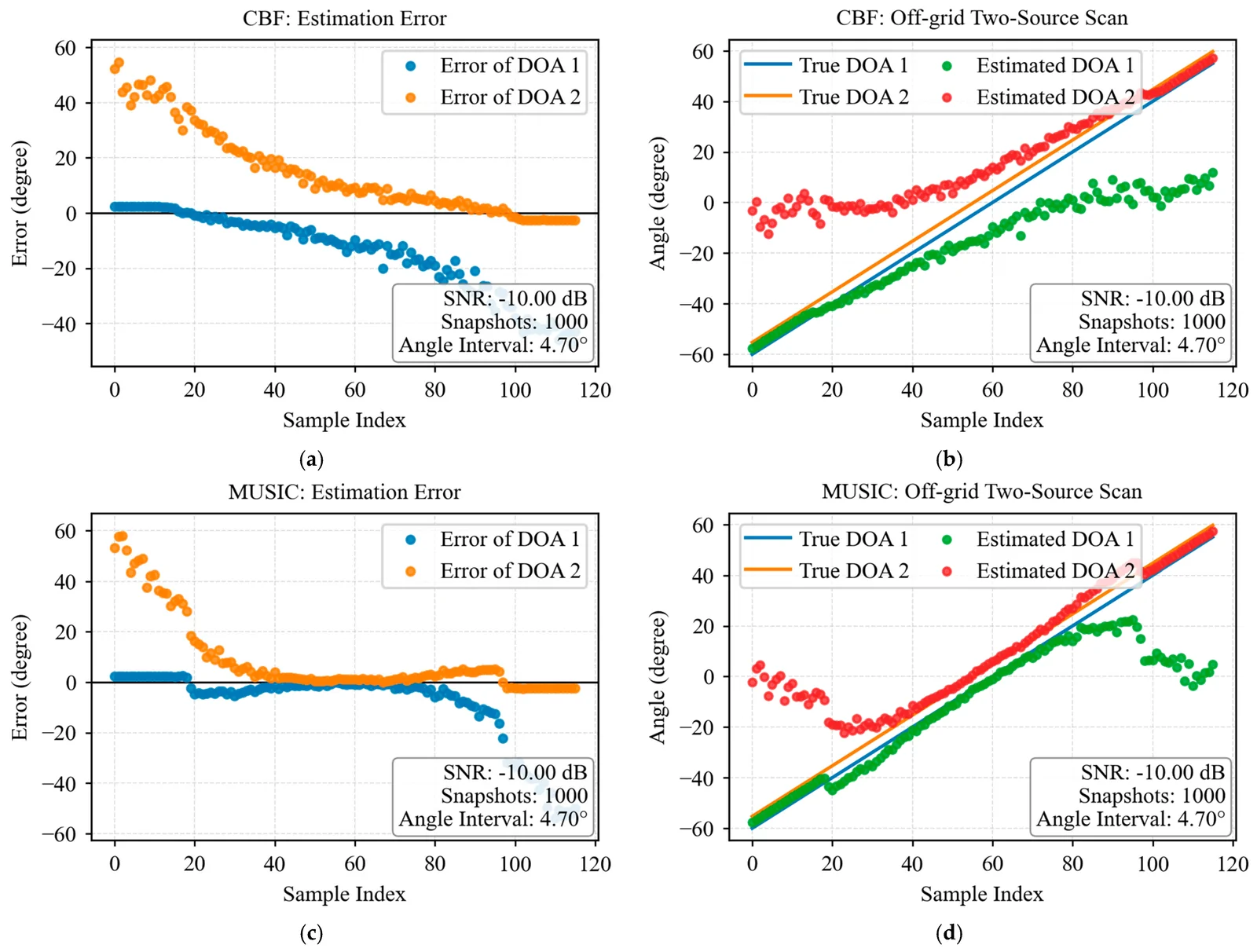

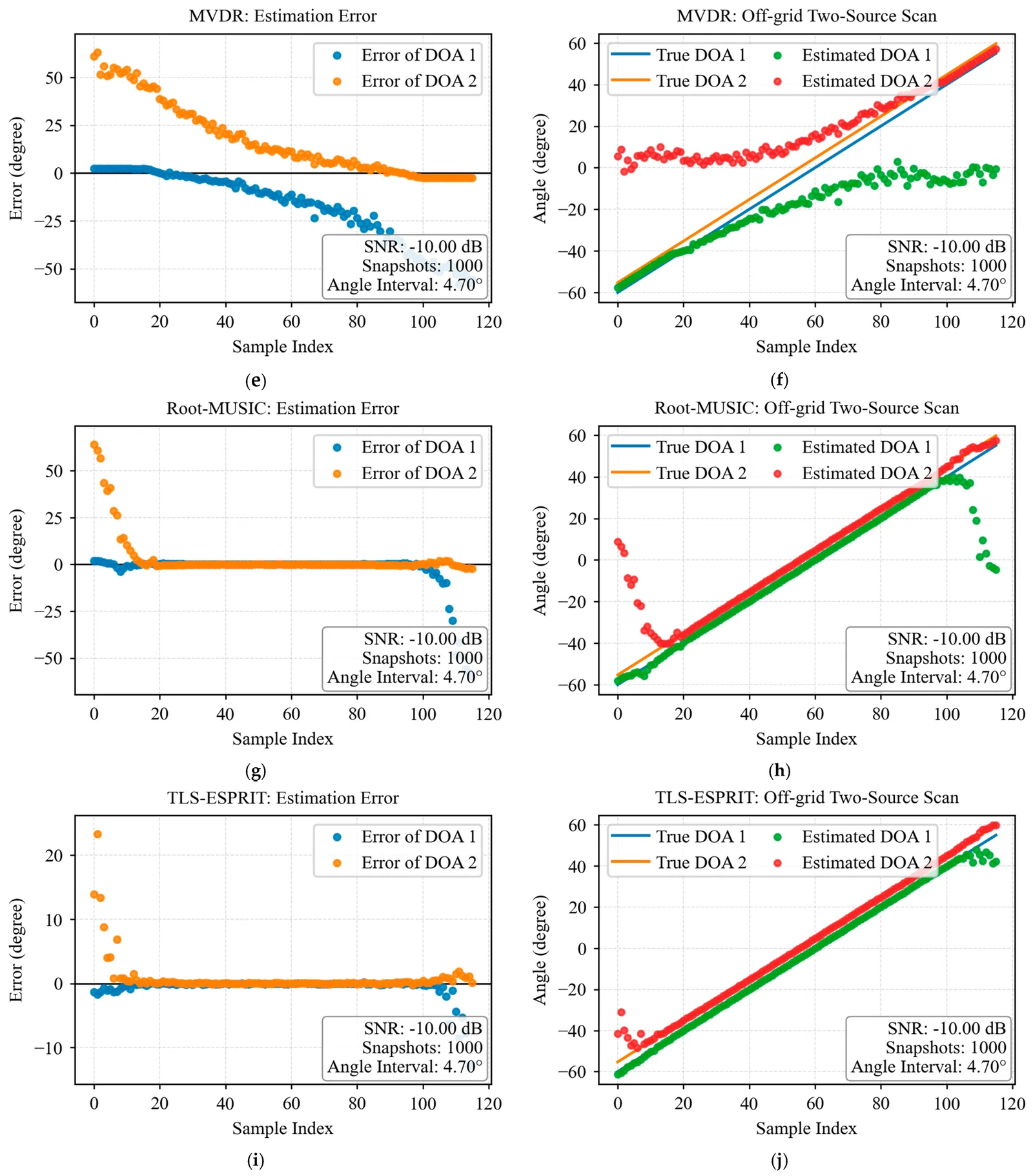

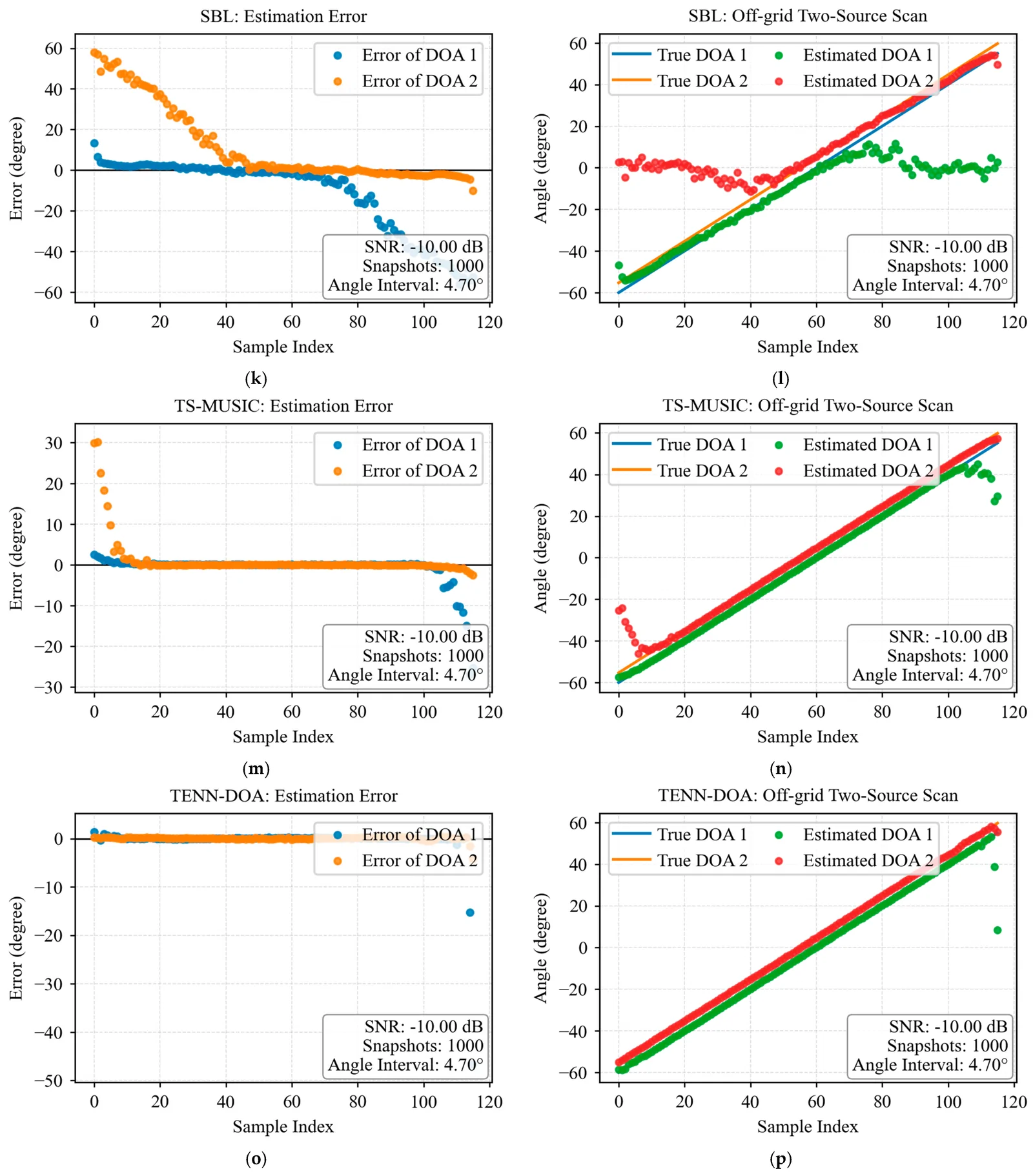
Scenario 1 (SNR = −10 dB; Δ*θ* = 4.7°; *N* = 1000; scan range: −60° to 55°). (**a**–**p**) Estimation errors and DOA trajectories for CBF, MUSIC, MVDR, Root-MUSIC, TLS-ESPRIT, SBL, TS-MUSIC, and TENN-DOA (two panels per method).

As shown in Figure 13,in Scenario 1, TENN-DOA produces a comparatively smooth trajectory and a concentrated error distribution over the scanned interval. MUSIC, CBF, and MVDR show more peak drift and missed detections near the boundaries; TLS-ESPRIT and Root-MUSIC also exhibit several larger errors.

#### 4.4.2. Scenario 2: Small Angular Separation with Limited Snapshots

In Scenario 2, the source separation is 2.11°, SNR = −5 dB, and *N* = 100. The separately trained [−60°, 60°], 1-grid model is used. This setting examines a small source separation with relatively few snapshots for a wide-angle profile.

**Figure 14:**
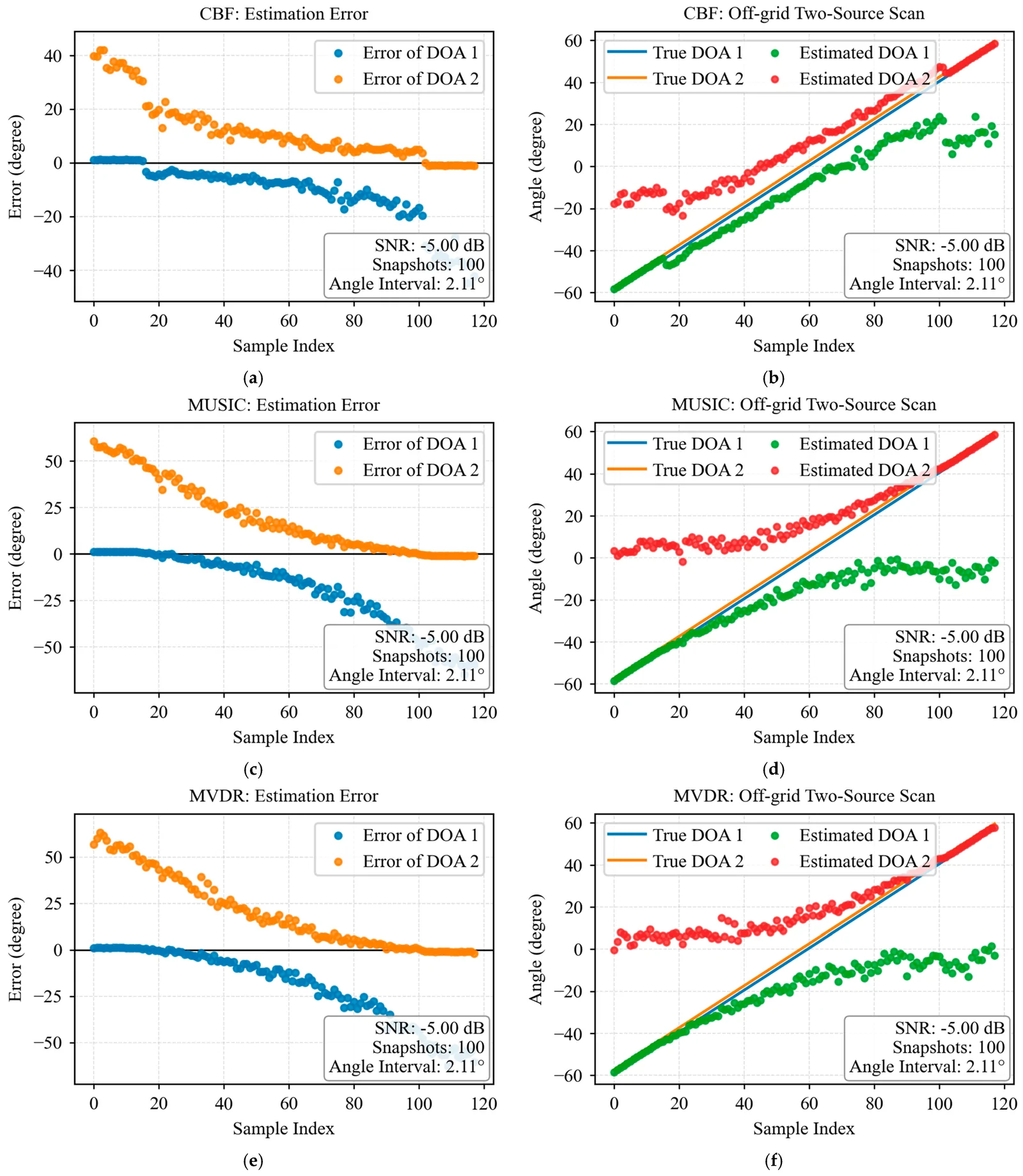

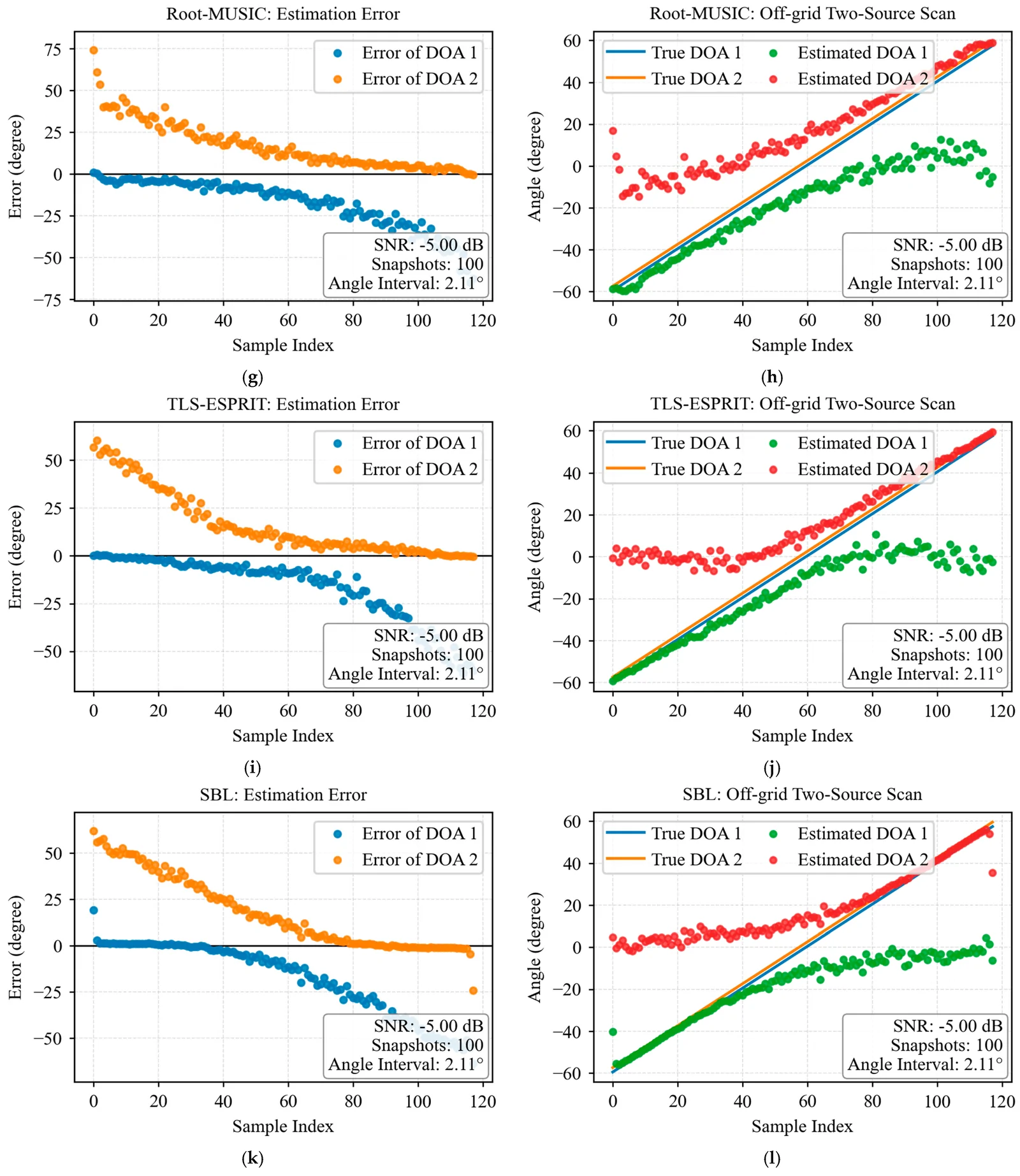

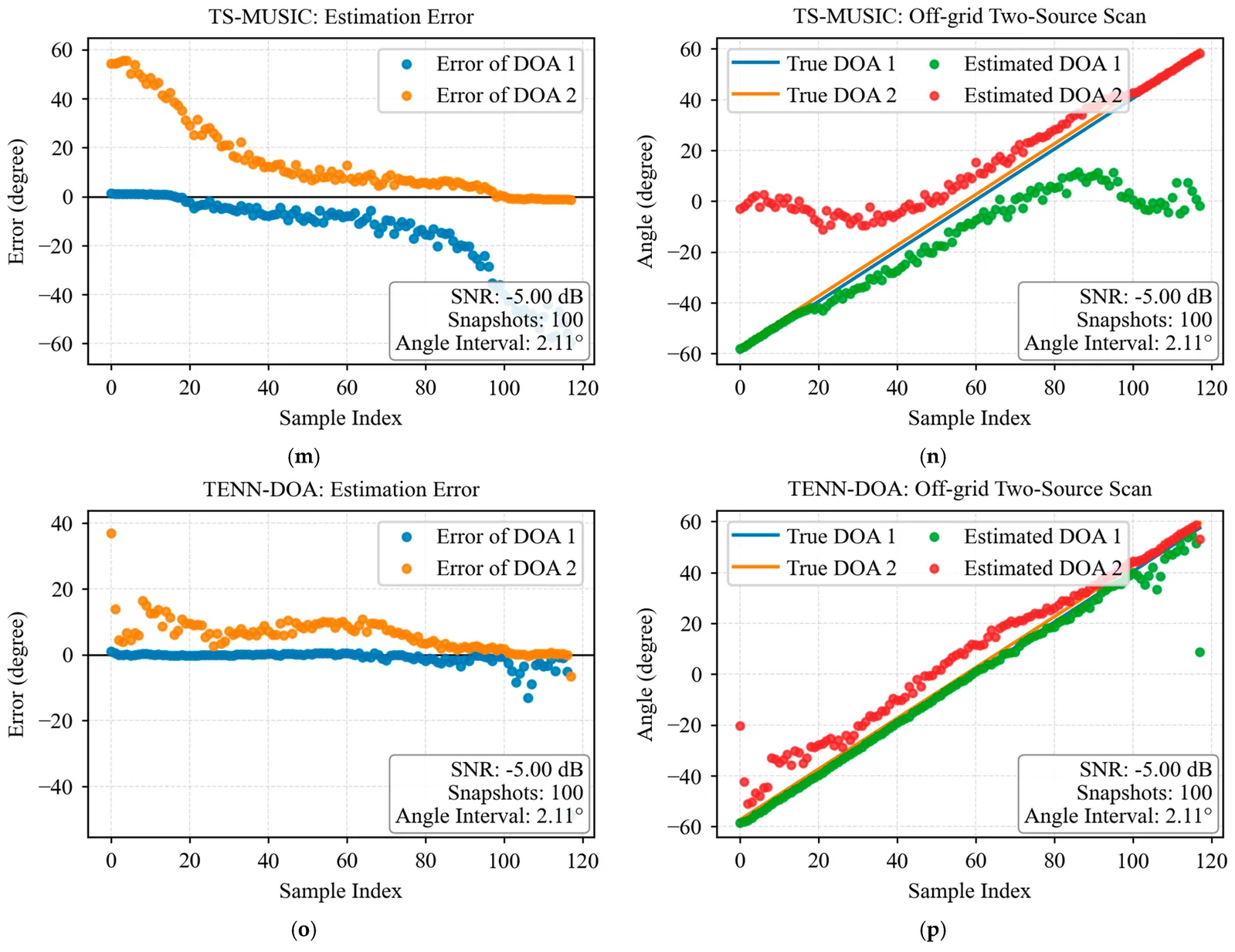
Scenario 2 (SNR = −5 dB; Δ*θ* = 2.11°; *N* = 100; scan range: −60° to 60°). (**a**–**p**) Estimation errors and DOA trajectories for CBF, MUSIC, MVDR, Root-MUSIC, TLS-ESPRIT, SBL, TS-MUSIC, and TENN-DOA (two panels per method).

As shown in Figure 14,in Scenario 2, TENN-DOA remains usable for the tested small separation and snapshot count, while MUSIC, Root-MUSIC, and TLS-ESPRIT show larger or more variable errors in parts of the scan. The sparse reconstruction methods are competitive at some angles and use their own algorithmic settings.

#### 4.4.3. Scenario 3: Wide Angular Domain and End-Fire Directions

In Scenario 3, the search domain is −90° to 90°, the source separation is 4.7°, SNR = −10 dB, and *N* = 1000. A separately trained *M* = 16 model with a 1° grid over [−90°, 90°] is used. The scan includes end-fire directions, where the steering vector sensitivity and boundary effects can increase peak distortion.

**Figure 15:**
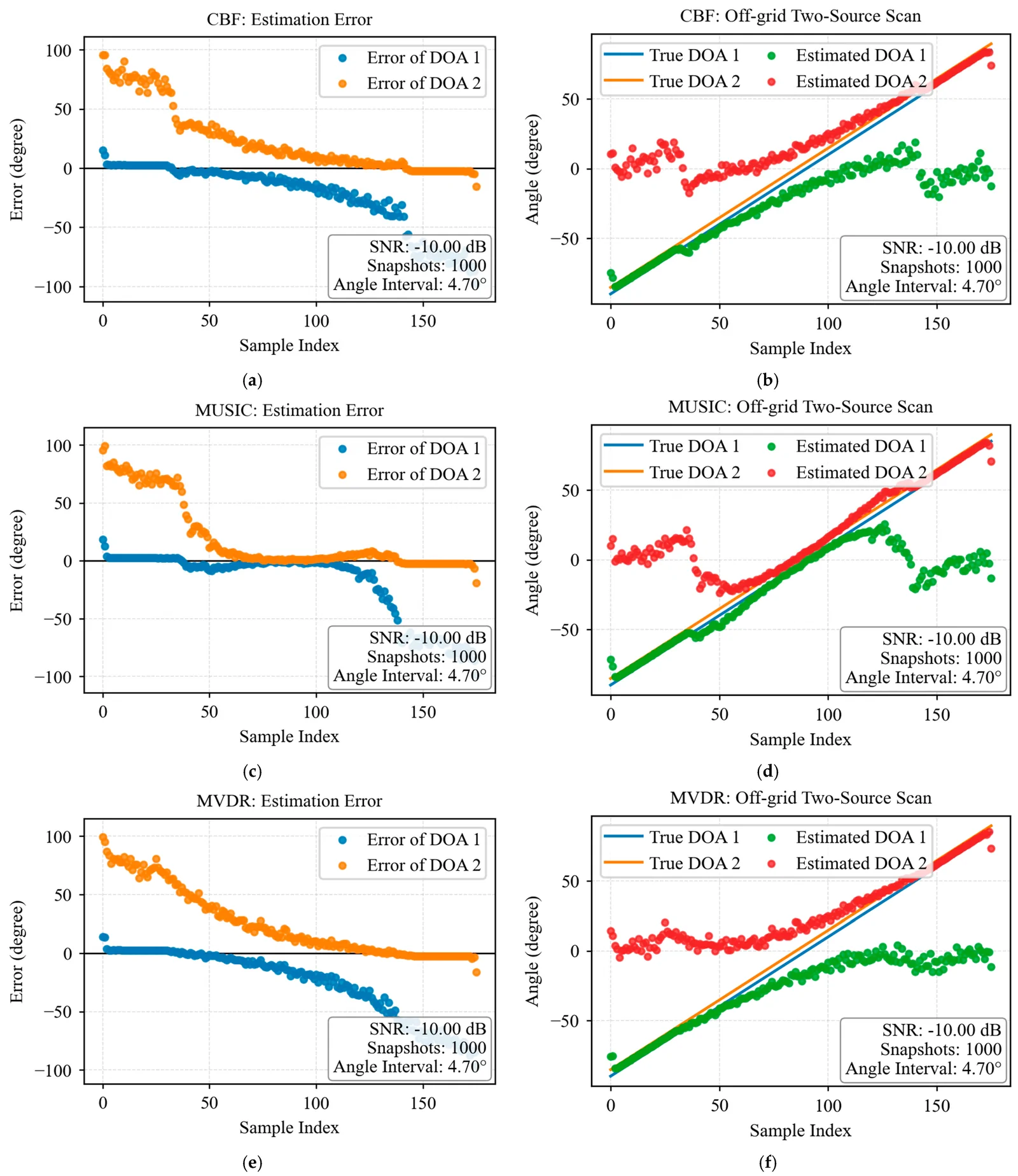

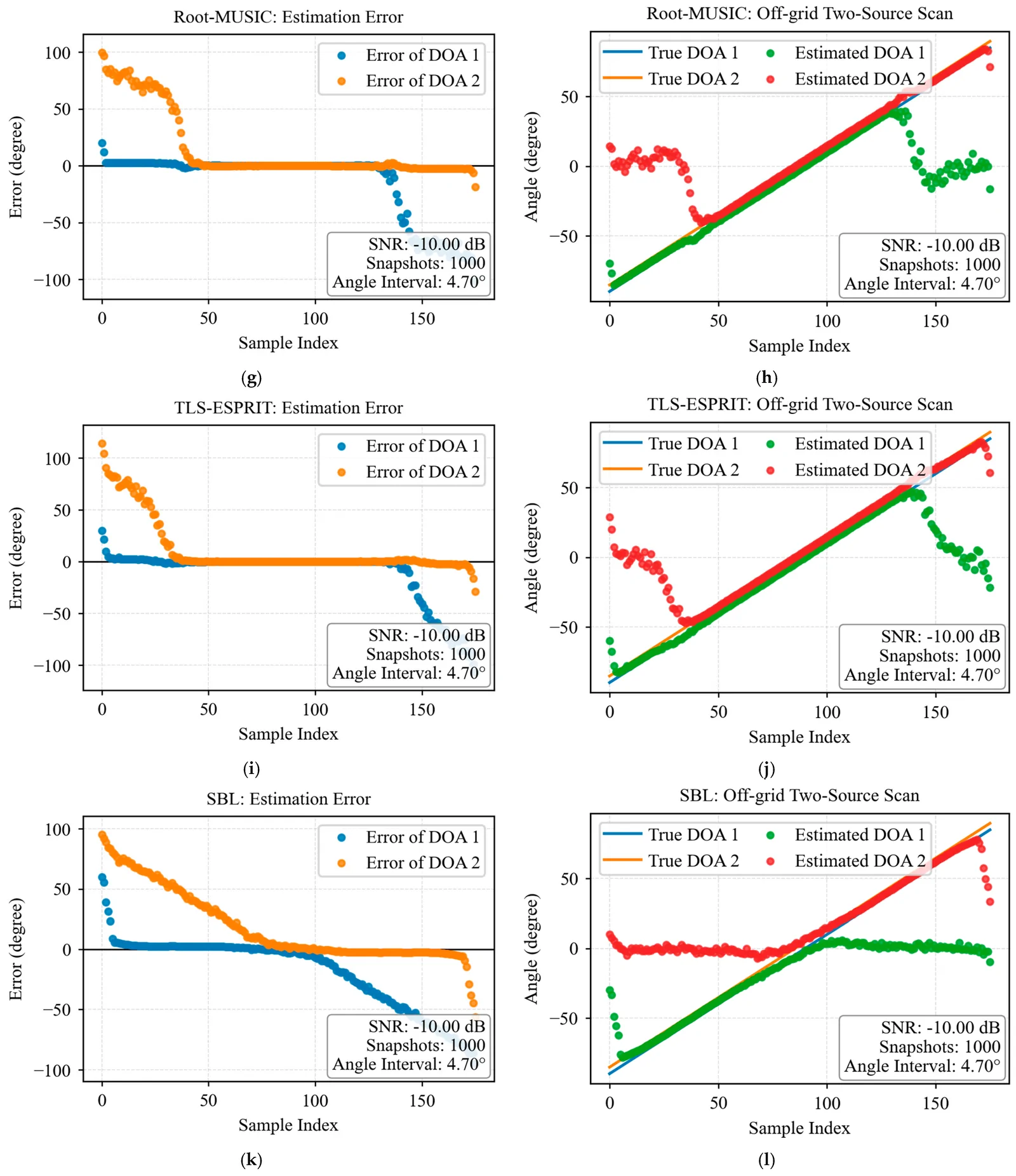

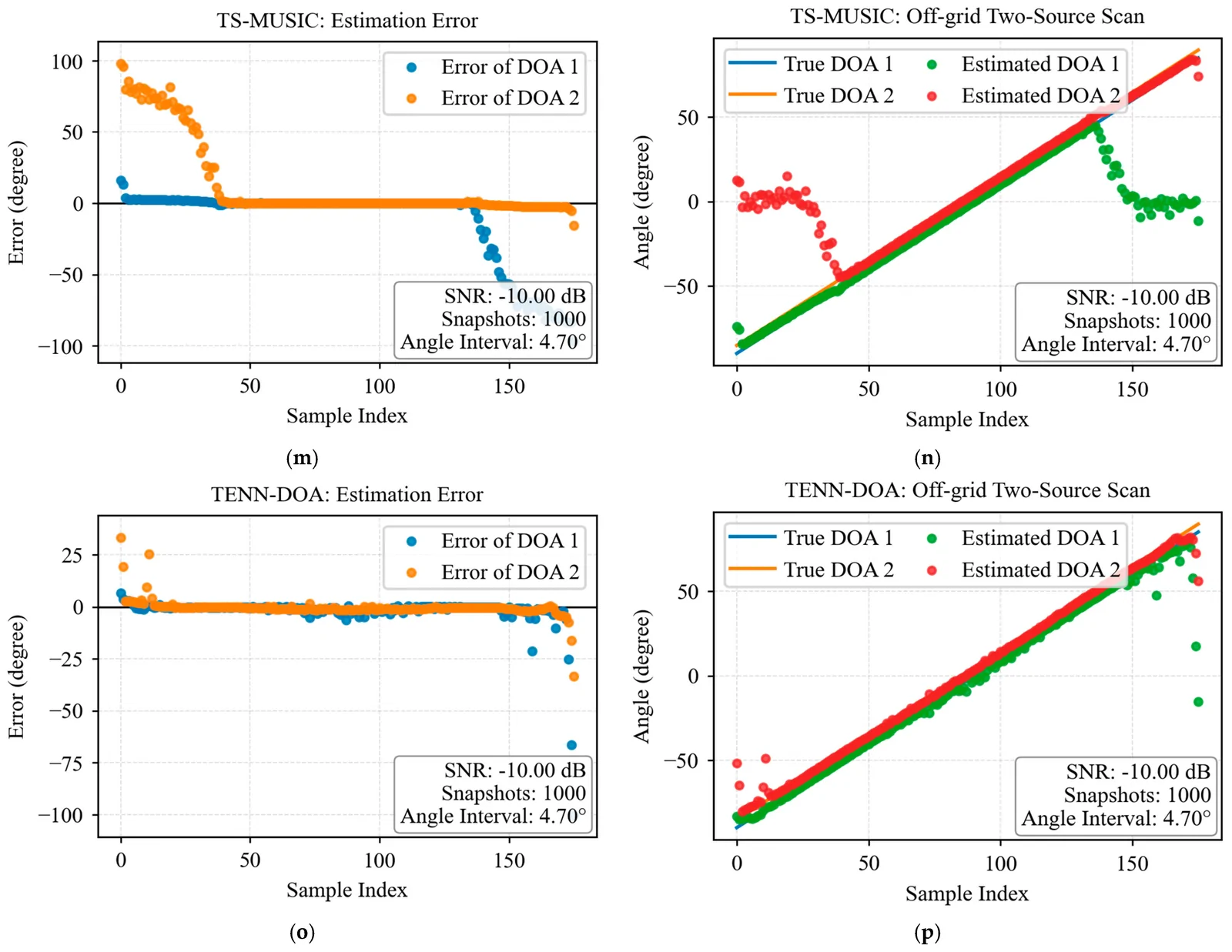
Scenario 3 (SNR = −10 dB; Δ*θ* = 4.7°; *N* = 1000; scan range: −90° to 90°, including end-fire). (**a**–**p**) Estimation errors and DOA trajectories for CBF, MUSIC, MVDR, Root-MUSIC, TLS-ESPRIT, SBL, TS-MUSIC, and TENN-DOA (two panels per method).

As shown in Figure 15, the results show that TENN-DOA maintains stability in the wide-angle domain and exhibits certain advantages near the end-fire direction. In contrast, the MUSIC, Root-MUSIC, and TLS-ESPRIT algorithms generally produce larger errors in the boundary regions, and severe mismatch occurs in some samples. This indicates that in the simultaneous presence of main-lobe broadening, boundary effects and noise perturbations, the structurally enhanced input features and local pattern discrimination capability of the convolutional network can better guarantee the continuity and accuracy of direction-of-arrival estimation results.

### 4.5. Discussion

The simulation results lead to two observations. First, structured covariance processing and angular matching improve the learned estimator in the low SNR, limited snapshot, two-source ULA regimes studied. Second, the gain is not attributable to network size alone: the controlled ablation separates the raw covariance input, covariance enhancement, angular matching, and receptive field schedule.

The study remains limited to a known *K* = 2, trained angular grids, and simulated ideal ULA data. Measured arrays, larger or unknown source counts, learned or perturbed dictionaries, multiple independently trained checkpoints, and continuous TENN localization remain open problems.

## 5. Conclusions

This paper proposes TENN-DOA, a grid-based hybrid estimator for ideally calibrated uniform linear arrays, where the scenario consists of two uncorrelated signal sources with equal power and spatially white complex Gaussian noise. The method integrates Hermite–Toeplitz projection, data-adaptive shrinkage, power spectral density projection, real-domain angle matching, and a lightweight one-dimensional convolutional neural network. Two grid-constrained directions-of-arrival are obtained via peak detection.

Major comparative simulations are conducted concerning the signal-to-noise ratio, snapshot number, angular separation and grid mismatch. The compared approaches include conventional beamforming, minimum variance distortion less response, multiple signal classification, root-multiple signal classification, total least-squares estimation of signal parameters via rotational invariance techniques, data-adaptive multiple signal classification, sparse Bayesian learning, off-grid sparse Bayesian learning, and TS-MUSIC. The simulation results demonstrate that the proposed method achieves a higher resolution probability and lower root mean square error under harsh conditions, such as low signal-to-noise ratio, limited snapshots and closely spaced signal source angles.

Future research can conduct evaluation and validation for a greater or unknown-number of signal sources, correlated signal sources with unequal power, real-world array calibration and coupling effects, colored non-Gaussian noise, learned or perturbed dictionaries, multiple training random seeds, and continuous localization and uncertainty-aware localization.

## Figures and Tables

**Table 1:** Comparison of related algorithms.

Approach	Structured Covariance	Learned Stage	Continuous Output	Role in Revision
Toeplitz reconstruction [10]	Yes	No	Estimator dependent	Related structured baseline
Sparse prior CNN [22]	Not the same front end	Yes	Grid based	Closest hybrid learning category
Complex gridless DNN [23]	Covariance reconstruction	Yes	Yes	Continuous learning comparator
DA-MUSIC [29]	Learned covariance enhancement	Yes	Grid/subspace	Learning-based baseline
TENN-DOA	Toeplitz + shrinkage	Yes	Grid based	Structured front end + angular matching + compact CNN

**Table 2:** TENN dataset and training configuration.

Type	Setting
Primary array/grid	*M* = 40; *d* = *λ*/2; [−6°, 6°]; Δ = 0.1°; *L* = 121; *K* = 2
Primary stored dataset	43,200 scenes; 34,560 train/8640 validation; *N* = 70; dataset and training seed 42
Primary training SNR	Uniform [−10, 10] dB
Primary loss/output	Two hot targets; MSE; sigmoid output; no class reweighting
Primary comparison CNN	Kernels 21/15/11/5/3; stride 1; same padding; hidden BN + ReLU; no pooling
Controlled ablation	43,200 shared scenes; 80/20 split; kernels 21/17/13/9/5 for A0; 150 epochs; dataset seed 1200; train seed 42; 300 independent test scenes per point; test seed base 880,000
Main comparison tests	500 independent scenes per operating point (10 repeats × 50); base seed 42 with deterministic Seed Sequence derivation
Wide angle [−60°, 60°]	*M* = 16; Δ = 1°; *L* = 121; 36,300 grid-pair scenes; separate model
Wide angle [−90°, 90°]	*M* = 16; Δ = 1°; *L* = 121; 81,450 grid-pair scenes; separate model
Wide-angle training	SNR ∈ {−20, −15, −10, −5, 0} dB; *N* ∈ {100, 200, 500, 1000}; BCE; seed 42
Peak detection	Known *K* = 2; local peaks separated by ≥5 bins; top two bin fallback
Baselines	CBF, MVDR, MUSIC, Root-MUSIC, TLS-ESPRIT, DA-MUSIC, SBL, off-grid SBL, TS-MUSIC

**Table 3:** Representative uncertainty summary for SNR = −10 dB, *N* = 100, and true DOAs [0°, 1°].

Method	RMSE (Deg), 95% CI	PoR, 95% CI	Test Scenes
TENN-DOA	0.315 [0.247, 0.379]	0.962 [0.941, 0.976]	500
SBL	0.877 [0.784, 0.971]	0.330 [0.290, 0.372]	500
Off-grid SBL	0.886 [0.791, 0.976]	0.378 [0.337, 0.421]	500
DA-MUSIC	2.438 [2.337, 2.535]	0.368 [0.327, 0.411]	500
MUSIC	2.736 [2.694, 2.778]	0.018 [0.009, 0.034]	500

**Table 4:** Ablation variants.

Variant	Model	Modules	Description
A0	Full TENN-DOA	Toeplitz + shrinkage + angular matching + 61-bin CNN	Complete controlled ablation model
A1	SCM–Direct CNN	Raw SCM real vector + CNN	Covariance enhancement and angular matching removed
A2	TS–Direct CNN	Toeplitz + shrinkage + direct covariance vector + CNN	Angular matching removed
A3	TS-AD-SCNN	Toeplitz + shrinkage + angular matching + 3-bin CNN	Small-kernel back end

## Data Availability

The data reported in this study are generated from numerical simulations and can be reproduced using the model parameters and simulation settings described in the manuscript. Additional simulation data are available from the corresponding author upon reasonable request.

## Abbreviations

1D CNN	One-Dimensional Convolutional Neural Network
CBF	Conventional Beamforming
CC BY	Creative Commons Attribution
CNN	Convolutional Neural Network
DA-MUSIC	Deep Augmented MUSIC
DFT	Discrete Fourier Transform
DOA	Direction of Arrival
DOF	Degrees of Freedom
ESPRIT	Estimation of Signal Parameters via Rotational Invariance Techniques
IoT	Internet of Things
MSE	Mean Squared Error
MUSIC	Multiple Signal Classification
MVDR	Minimum Variance Distortion Less Response
PSD	Positive Semidefinite
PoR	Probability of Resolution
RMSE	Root Mean Square Error
Root-MUSIC	Root-Multiple Signal Classification
SCM	Sample Covariance Matrix
SNR	Signal-to-Noise Ratio
SVM	Support Vector Machine
TENN-DOA	Toeplitz-Enhanced Neural Network for Direction-of-Arrival Estimation
TLS-ESPRIT	Total Least-Squares ESPRIT
TS-MUSIC	Toeplitz-Shrinkage MUSIC
UAMP	Unitary Approximate Message Passing
ULA	Uniform Linear Array

## References

[1] Krim H., Viberg M. (1996). Two decades of array signal processing research: The parametric approach. IEEE Signal Process. Mag..

[2] Pesavento M., Trinh-Hoang M., Viberg M. (2023). Three more decades in array signal processing research: An optimization and structure exploitation perspective. IEEE Signal Process. Mag..

[3] Schmidt R.O. (1986). Multiple emitter location and signal parameter estimation. IEEE Trans. Antennas Propag..

[4] Roy R., Kailath T. (1989). ESPRIT-estimation of signal parameters via rotational invariance techniques. IEEE Trans. Acoust. Speech Signal Process..

[5] Stoica P., Nehorai A. (1989). MUSIC, maximum likelihood, and Cramer-Rao bound. IEEE Trans. Acoust. Speech Signal Process..

[6] Griffiths H.D., Baker C.J. (2005). Passive coherent location radar systems. Part 1: Performance prediction. IEE Proc.-Radar Sonar Navig..

[7] Richmond C.D. (2006). Mean-squared error and threshold SNR prediction of maximum-likelihood signal parameter estimation with estimated colored noise covariances. IEEE Trans. Inf. Theory.

[8] Ledoit O., Wolf M. (2004). A well-conditioned estimator for large-dimensional covariance matrices. J. Multivar. Anal..

[9] Chen Y., Wiesel A., Eldar Y.C., Hero A.O. (2010). Shrinkage algorithms for MMSE covariance estimation. IEEE Trans. Signal Process..

[10] Dai J., Qiu T., Luan S., Tian Q., Zhang J. (2023). An Improved Toeplitz Approximation Method for Coherent DOA Estimation in Impulsive Noise Environments. Entropy.

[11] Yang Z., Xie L. (2016). Enhancing sparsity and resolution via reweighted atomic norm minimization. IEEE Trans. Signal Process..

[12] Liu L., Rao Z. (2022). An Adaptive Lp Norm Minimization Algorithm for Direction of Arrival Estimation. Remote Sens..

[13] Sun P., Fu Y., Guo Q., Zou K., Gong K., Wang W. (2024). A low-complexity DOA estimation algorithm using UAMP with Bernoulli-Gaussian prior. Digit. Signal Process..

[14] Pal P., Vaidyanathan P.P. (2010). Nested arrays: A novel approach to array processing with enhanced degrees of freedom. IEEE Trans. Signal Process..

[15] Vaidyanathan P.P., Pal P. (2011). Sparse sensing with co-prime samplers and arrays. IEEE Trans. Signal Process..

[16] Li R., Tian D., Liang Z., Chang S., Liu Q. (2025). Multi-frequency based underdetermined direction-of-arrival estimation for irregularly positioned sparse array. Digit. Signal Process..

[17] Yu J., Xu Y., Zhang S., Liu Z. (2024). Near-field DOA estimation oriented sparse array design via symmetric augmentation of three ULAs. Digit. Signal Process..

[18] Liu Z.-M., Zhang C., Yu P.S. (2018). Direction-of-arrival estimation based on deep neural networks with robustness to array imperfections. IEEE Trans. Antennas Propag..

[19] Papageorgiou G.K., Sellathurai M., Eldar Y.C. (2021). Deep networks for direction-of-arrival estimation in low SNR. IEEE Trans. Signal Process..

[20] Zhang Y., Dou H. (2025). A novel hierarchical classification for DoA estimation using coprime array with sensor location errors. Digit. Signal Process..

[21] Jiang Y., Liu F. (2024). Adaptive Joint Carrier and DOA Estimations of FHSS Signals Based on Knowledge-Enhanced Compressed Measurements and Deep Learning. Entropy.

[22] Wu L.-L., Liu Z.-M., Huang Z.-T. (2019). Deep convolution network for direction of arrival estimation with sparse prior. IEEE Signal Process. Lett..

[23] Cao Y., Zhou T., Zhang Q. (2024). Gridless DOA Estimation Method for Arbitrary Array Geometries Based on Complex-Valued Deep Neural Networks. Remote Sens..

[24] Guo S., Zhang Q., Fu X., Zheng G., Zhou H. (2025). DOA estimation method for sparse arrays based on deep convolutional autoencoder and deep convolutional neural network. Digit. Signal Process..

[25] Chen K.-L., Rao B.D. (2025). Subspace representation learning for sparse linear arrays to localize more sources than sensors: A deep learning methodology. IEEE Trans. Signal Process..

[26] Huang Q., Fang W. (2022). DOA estimation using two independent convolutional neural networks with residual blocks. Digit. Signal Process..

[27] Xiao L., Zheng G., Song Y., Zheng H. (2025). Real-time and high-precision SVM-RF based prediction model for DOA estimation. Digit. Signal Process..

[28] Wang K., Yu C., Zhong F., Yu Z. (2025). Accurate DFT-based DOA estimation of multi sources in uniform linear array. Digit. Signal Process..

[29] Merkofer J.P., Revach G., Shlezinger N., Routtenberg T., van Sloun R.J.G. (2024). DA-MUSIC: Data-driven DoA estimation via deep augmented MUSIC algorithm. IEEE Trans. Veh. Technol..

[30] Zhang B., Yuan S. (2022). Shrinkage estimators of large covariance matrices with Toeplitz targets in array signal processing. Sci. Rep..

